# Pilot Implementation of a User-Driven, Web-Based Application Designed to Improve Sexual Health Knowledge and Communication Among Young Zambians: Mixed Methods Study

**DOI:** 10.2196/37600

**Published:** 2022-07-07

**Authors:** Anjali Sharma, Chanda Mwamba, Mwila Ng'andu, Vikwato Kamanga, Mayamiko Zoonadi Mendamenda, Yael Azgad, Zainab Jabbie, Jenala Chipungu, Jake M Pry

**Affiliations:** 1 Centre for Infectious Disease Research in Zambia Lusaka Zambia; 2 Avert London United Kingdom; 3 Department of Public Health Sciences University of California Davis, CA United States

**Keywords:** sexual and reproductive health, web application, digital health intervention, pilot study, quasi-experiment, adolescent, young people, Zambia, sub-Saharan Africa, mobile phone

## Abstract

**Background:**

Digital health interventions show promise in improving the uptake of HIV services among adolescents and young people aged 15 to 24 years in sub-Saharan Africa.

**Objective:**

This study aimed to pilot-test a theory-based, empirically grounded web-based application designed to increase condom-related knowledge, sexual and reproductive health (SRH) communication, and healthier choices among young Zambians.

**Methods:**

We conducted a pre-post quasi-experimental evaluation of the user-driven *Be in the Know Zambia* (BITKZ) web application using web-based surveys and in-depth interviews (IDIs) on the phone. We enrolled participants using social media advertisements. Our final analysis set comprised 46.04% (749/1627) of participants in the intervention group (which received the BITKZ link) and 53.96% (878/1627) of participants in the comparison group (no intervention). We collected survey data at study enrollment (baseline) and 5 weeks after the first enrollment in each group. Approximately 85% (637/749) of BITKZ users completed a user survey, of whom 9.3% (59/637) participated in IDIs. We calculated the time interfacing with BITKZ using the application log files. We conducted descriptive analyses to describe baseline characteristics and the user experience. At the endline, we assessed association using a *t* test and adjusted logistic regression for binary outcomes and ordinal regression for ordered outcomes, conditioning on age, sex, marital status, and employment status. We used adjusted average treatment effects (aATE) to assess the effects of BITKZ intervention. We conducted rapid matrix analyses of IDI transcripts in Microsoft Excel, sorting the data by theme, gender, and experience rating.

**Results:**

Users rated BITKZ highly (excellent: 352/609, 57.8%; good: 218/609, 35.8%). At the endline, the intervention group had a higher level of knowledge related to condoms (adjusted odds ratio [aOR]: 1.35, 95% CI 1.06-1.69) and on wearing condoms correctly (aOR: 1.23, 95% CI 1.02-1.49). Those who had full-time employment had increased odds of knowing how to wear condoms correctly (aOR: 1.67, 95% CI 1.06-2.63) compared with those who reported being unemployed, as did men when compared with women (aOR: 1.92, 95% CI 1.59-2.31). Those in the intervention group were more likely to score higher for intention to test for sexually transmitted infections (STIs; aATE 0.21; *P*=.01) and HIV (aATE 0.32; *P*=.05), as well as for resisting peer pressure (aATE 2.64; *P*=.02). IDIs corroborated increased knowledge on correct condom use among men and female condoms among women, awareness of STIs and testing, and resistance to peer pressure. Interviewees provided examples of more open SRH communication with partners and peers and of considering, adopting, and influencing others to adopt healthier behaviors.

**Conclusions:**

Despite the high baseline awareness of SRH among Zambian adolescents and young people with internet access, BITKZ provided modest gains in condom-related knowledge, resistance to peer pressure, and intention to test for STIs and HIV.

## Introduction

The current rate of decline in HIV incidence among adolescents and young people aged 15 to 24 years is insufficient to end the AIDS epidemic by 2030 [1]. Southern and East African regions, although performing well, are projected to achieve an 84% reduction in 2050 from 2010 HIV incidence rates among adolescents and young people [1], which is below the 90% reduction target for 2030 [2]. Although overall HIV incidence has decreased in these regions, disproportionate numbers of urban young women aged 15 to 24 years and young men aged 20 to 29 years [3] are newly infected with HIV, albeit with some country-level variations [4]. The COVID-19 pandemic further complicated health service delivery and threatens to reverse decade-long gains in HIV prevention among adolescents and young people in sub-Saharan Africa (SSA) [5,6].

Digital health interventions (DHIs) offer an opportunity to reach digitally connected adolescents and young people with sexual and reproductive health (SRH) information, education, and services during the COVID-19 pandemic restrictions [7,8]. An age-unrestricted meta-analysis of SSA data found that DHIs improved HIV prevention knowledge and intention to act but not attitudes or perceived self-efficacy [9]. In addition, DHIs without human interaction showed no effect on the uptake of HIV prevention behaviors [9]. Interactive DHIs provide knowledge and tailored personalized feedback to support emotions, decision-making, and behavior change. Another meta-analysis, including a Zambian and Ugandan study and 12 studies among adolescents and young people, found positive effects of interactive DHIs on HIV prevention knowledge, intention, and behavior [10]. Finally, a meta-analysis of SRH DHI among adolescents and young people showed increased condom use, reduced sexual intercourse, and mixed results for improved knowledge but did not include any studies from SSA [11]. These mixed and limited results suggest the need for more research on the effect of DHIs on SRH among adolescents and young people across SSA [8,9,11].

Although a comparatively low proportion of adolescents and young people live with HIV in Zambia [12], HIV prevalence has increased among urban young men aged 15 to 24 years [13], and young women aged 17 to 19 years remain at a precipitous risk for new HIV infections [14]. Progress toward reducing HIV incidence in this population remains suboptimal [15]. The COVID-19 pandemic has further disrupted government-endorsed, school-based, and interpersonal structural, behavioral, and combination HIV prevention efforts and may have increased reliance on mass and social media platforms [15-18]. We sought to add to these efforts by designing a user-driven DHI incorporating informational elements based on the expressed needs and preferences of adolescents and young people during the containment periods of the first 2 COVID-19 waves in Zambia [19]. In this paper, we present the effect of the pilot implementation of the cocreated *Be in the Know Zambia* (BITKZ) web-based application on SRH knowledge, communication, intentions, and behavior. This study adds to the growing body of literature on the measurement of the effect of DHI on SRH among adolescents and young people in SSA [9,20-23].

## Methods

We conducted a pre-post quasi-experimental mixed methods evaluation using web-based survey data collection on Qualtrics (Qualtrics International Inc) [24] and in-depth interviews (IDIs) on the phone from June to August 2021.

### Enrollment

To target persons eligible for the pilot study, we placed advertisements on Facebook (Meta Platforms Inc) and contacted established Zambian adolescent groups in youth-friendly SRH spaces identified in formative phase interactions and followed up using WhatsApp (Meta Platforms Inc) groups or email. WhatsApp is a commonly used mobile-based SMS text message app. Individuals aged 18 to 24 years, living in Zambia, and able to understand and give informed consent in English (the language of the app) were eligible. Those who did not meet all the eligibility criteria or declined to consent were excluded.

Eligible individuals were required to provide consent for data storage and analyses for publication purposes and to be contacted by phone or email. In addition, those interested in participating in the study were required to provide a phone number or email address to receive a link to a secure baseline and endline survey and to receive airtime on completing each survey.

The first 1500 individuals were targeted for the intervention group and were consecutively enrolled if they were eligible and agreed to join the BITKZ application for at least 1 month and complete the user and endline surveys. The next 1500 individuals were consecutively enrolled into the comparison group if they were eligible and agreed to participate in the endline survey after a month of enrollment. We decided to enroll adolescents and young people sequentially rather than in parallel to ensure an adequate sample size in the intervention arm, given that there was no precedent for this type of web-based enrollment, intervention delivery, and evaluation in Zambia.

Those enrolled in the intervention group were also offered a chance to be invited to an IDI on either (1) their user experience and interaction with the application or (2) their user experience with each feature of the BITKZ app. Individuals who agreed to be contacted for an IDI were asked for their preferred contact information and consent to audio recording and transcription.

### Intervention

Avert, a UK-based organization, engineered the BITKZ internet or web-based application to provide SRH content for young Zambians aged 18 to 24 years. Avert uses innovative digital health approaches to help adolescents and young people make healthier SRH choices to reduce adverse SRH outcomes and ultimately improve their lives. The application content and design features were based on insights from the formative phase from August 2020 to May 2021, including qualitative phone-based interviews with 18 Zambian adolescents and young people from August to October 2020 and cocreation sessions held with 51 Zambian adolescents and young people from September to December 2020 on Facebook (3 groups of 7 men, 9 women, and 6 both) and WhatsApp (3 groups of 9 men, 7 women, 13 both). These groups further helped refine the BITKZ functionality through user feedback collected between March and May 2021.

BITKZ is grounded in the practical application of theory-based methods [25] and builds on the foundational work collected during the initial formative phase. During the formative work, we discovered that adolescents and young people did not know how to use condoms, had unplanned sex under the influence of peers, and longed to receive guidance from knowledgeable and trusted sources. Thus, BITKZ provided SRH information to adolescents and young people to increase condom-related knowledge, resistance to peer pressure, and SRH communication with people who matter to support the outcomes of interest to Avert—intention and practice to prevent sexually transmitted infections (STIs), HIV, and unintended pregnancies. Multimedia Appendix 1 demonstrates how BITKZ used the taxonomy provided by Kok et al [25] to map the intervention to the behavior we intended to change using levers that responded to the formative work while being pragmatic because of the limited time, resources, and communication platforms available for the project. BITKZ tailored SRH information to include imagery, language, and context relevant to the adolescents and young people in Zambia, setting up scenario-based risk information to encourage dialog, active learning, and social support. These scenarios, along with positive and gain-based messages, aimed to promote implementation intentions and goal setting.

**Figure 1:**
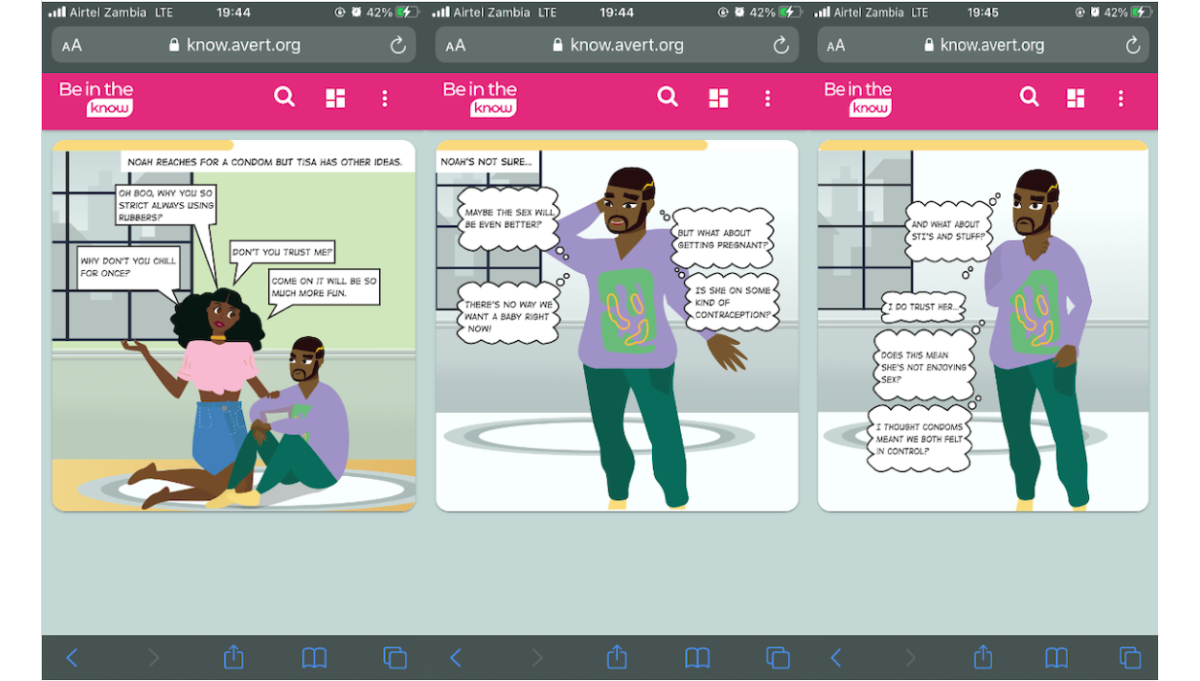
Screenshot of the scenario.

In application, BITKZ appealed to personal identity [26] by inviting players to choose among 3 male and 3 female characters aged 18 to 24 years, with distinctive traits (modern independent go-getters, sophisticated dreamers, or religious loners) developed from narratives gathered during the formative phase of development. Users would then enter a comic strip where each male and female character faces dilemmas centered on 3 themes—condoms, preventing pregnancy, and staying healthy—aiming to provoke dialog and reflection [27]. Users could interact with these 3 themes through 6 features, the screenshots of which are depicted in Multimedia Appendix 2 to illustrate how they might aid adolescent and young people users in SRH-related communications. Briefly, the 6 features included the comic strips modeling dilemmas using real-life scenarios described previously (Figure 1), visual guides on how to use various condoms and contraceptives, sharable *Let’s Talk* and *Top Tips* cards to support users to think through choices and provide action-focused ideas, frequently asked questions about SRH, and quizzes.

These features aimed to positively reinforce knowledge and conversations exploring choices on topics that concern the user [28]. In addition, the application had a gamification feature [29] where users could win up to 5 web-based badges for commenting, sharing their favorite *Top Tips* and *Let’s Talk cards* using BITKZ’s integrated share function, looking for answers to frequently asked questions, reading all the visual guides, and completing all quizzes [30]. Given the variety of media with which participants might interact, we sought here only to assess any uptake compared with no uptake of or access to BITKZ materials.

Following the baseline survey on Qualtrics [24], participants in the intervention group could follow the application content in any order and frequency on any electronic device with an internet connection during the 3-month intervention period.

### Data Collection

#### Web-based Pre-Post Intervention Surveys

All surveys were developed and administered on Qualtrics [24], and reimbursements (airtime) were made through cGrate (Zambia Ltd) [31]. All participants enrolled and self-administered the baseline web-based survey from June 7 to August 3, 2021. On completion, they received 30 Zambian Kwacha (ZMW; US $1.50). Intervention participants who completed the baseline survey received a single-user link to the BITKZ application on email or WhatsApp (Meta Platforms Inc) as per their preference. Participants in the comparison group were advised to seek SRH advice as usual. Participants in the intervention group received a link to a self-administered web-based user survey a week after accessing the application. Those who completed the user surveys received 20 ZMW (US $1.00).

Intervention and comparison participants received an invitation 5 weeks after enrollment to complete the endline survey followed by biweekly reminders by WhatsApp (Meta Platforms Inc) or email. All participants self-administered the endline survey on the web from July 12 to September 2, 2021. On completion, they received 50 ZMW (US $2.50).

#### Phone IDIs

From July to August 2021, we conducted phone interviews with 59 participants who completed the endline and user surveys (Table 1).

We sequentially telephoned participants from among those who rated the BITKZ application as excellent, good, and poor, ensuring a gender balance. We interviewed 20% (12/59) of individuals each among those who rated the BITKZ application as *good* and *excellent* (replacing 2 individuals who missed 2 consecutive appointments). Of the 9 individuals who rated the application as *poor*, 8 (89%) agreed to the interview. We sequentially telephoned additional intervention participants to collect feedback on each application feature. Of the 78 participants who were telephoned, we interviewed a total of 27 (35%; n=13, 48% women, and n=14, 52% men) intervention participants on application features that stood out prominently for them to obtain at least six views on each feature. The remaining 86% (51/59) of participants did not answer, could not recall a feature, or recalled a feature already discussed by 6 interviewees.

We confirmed the identity of the person on the phone by name and age, after which we further asked whether they remembered being enrolled and interacting with the BITKZ application. If not, we thanked them for their time and went on to the next person on our list. We reminded others about their web-based agreement and confirmed their consent to the IDI, audio recording, and transcription. All interviews explored the effects of the application on their sexual health knowledge, communication, and behavior. Interviewees received 100 ZMW (US $5.00) reimbursement by cGrate [31].

**Table 1:** Postintervention in-depth interviews on BITKZ^a^ perception and experience (N=59).

Criteria		Men, n (%)	Women, n (%)	Total, N
**BITKZ rating**				
	Excellent	6 (50)	6 (50)	12
	Good	6 (50)	6 (50)	12
	Poor	4 (50)	4 (50)	8
Prominent features		14 (52)	13 (48)	27

^a^BITKZ: Be in the Know Zambia.

#### Survey Instruments and Measures

All measures were collected from the intervention and comparison groups using the same survey instrument at baseline and endline, which can be found in Multimedia Appendix 3.

The primary outcomes for all participants included condom-related knowledge, ability to talk to people who matter, and frequency of seeking SRH advice from them in the past month. Condom knowledge included 6 items (true or false) and the identification of 6 correct steps when using a condom from a list of 14 options adapted from Stanton et al [32]. Correct responses were coded as 1, and all other responses were coded as 0 to derive the mean and percentage of correct responses. The ability to seek advice on SRH included a list of 10 potential options for people who matter in the respondent’s life and was measured on a Likert scale ranging from definitely, probably, probably cannot, and definitely cannot (Cronbach α=.80) [33]. The frequency of seeking SRH advice by source in the past for month more than once a week, once a week, 1-2 times a month, and never) was calculated per source (Cronbach α=.86).

Other secondary outcomes measured the intention to use condoms at the next sexual intercourse (not at all, somewhat, and very likely). For a subpopulation of sexually active participants, we also asked about the intention to test for STIs if symptomatic and for HIV in the next 6 months. Higher scores reflected higher occurrence or likelihood of events.

We also collected participant characteristics, including self-reported age, gender, marital status, education level, perceived socioeconomic status relative to their community (rich-poor and very-not respected), and employment status. We asked about sexual debut (never had sex; true or false), currently sexually active (past 6 months; true or false), condom use at the last sexual intercourse (true or false), unplanned last sexual intercourse (true or false) [34], and most recent tests for STIs and HIV (<3 months, 3-6 months, 6-12 months, >12 months, and never). The condom knowledge questions correspond to questions 3.1 to 3.6 (Multimedia Appendix 3).

Other independent variables were drawn from sociocognitive theories [27] to include scales to measure permissive attitude (Cronbach α =.68), sexual norms (Cronbach α=.60), and self-efficacy (Cronbach α=.80), derived from a study by Muhammed et al [35] (Multimedia Appendix 3, question 4); frequency of being pressured into making unhealthy choices (every time, sometimes, rarely, and never) derived from the Kaiser National survey (Multimedia Appendix 3, question 5; Cronbach α=.80) [36]; and confidence to get a condom and STI or HIV test whenever wanted (very, somewhat, and not at all confident) [37]. Although we did not expect to see changes in these variables, we considered them as possible influencers of variables measuring intention and practice.

#### Application Experience and Use

We gathered ratings of overall participant application experience (excellent, good, and poor), as well as perceived aesthetics; engagement; functionality; reaction to the information; and the likelihood of sharing, continuing to use, and willingness to pay from the user survey (very, quite, and not at all).

The app software automatically generates a user log file that records each user interaction, which is time-stamped. These data were downloaded from the application’s website using Amplitude and the Amplitude export application programming interface.

#### Topics Covered by IDIs

Interviews with the first 32 participants explored the overall user experience, how the application content was used or had influenced the user, and recommendations for improvement. The remaining 27 IDIs explored experience with each of the six features: ease, aesthetics, entertainment, learning, sharing, change in SRH practices, and recommendations for improvement.

### Data Analysis

#### Quantitative Analysis

We conducted descriptive analyses to determine intervention status comparability and assess differences in participants’ sociodemographic characteristics, SRH norms, SRH behaviors, and communication about relationships and SRH, as well as to describe user experience.

We calculated a regression-adjusted average treatment effect (aATE) on SRH knowledge, permissive attitude, sexual norms, self-efficacy, peer pressure, and confidence in procuring STI tests and condoms, as well as intention to test for STI and HIV and use condoms at the next intercourse. Scores for permissive attitudes and sexual norms were based on the sum of positive SRH-competent responses to question sets (Multimedia Appendix 3, question 4). Statistical significance of differences was assessed using a *t* test, comparing mean differences between comparison and intervention at the endline, where appropriate. Regression analysis was used to assess the impact of BITKZ on condom-related knowledge. All multivariate impact analyses incorporated an intention-to-treat approach and controlled for sociodemographic characteristics (age, sex, marital status, educational attainment, employment status, perceived wealth, and perceived respectability at baseline), baseline measures of outcomes, and intervention status. All primary outcome analyses were completed using Stata (version 16.1) [38], and the duration per user per day was calculated using R software and the *dplyr* package [39].

#### Application Experience and Use

We analyzed participants’ use to assess the median time spent on the application. We gathered and analyzed the participants’ application experience (excellent, good, and poor) from the user survey.

#### Phone IDIs

We conducted a rapid matrix analysis [40] of IDIs to extract the overall user experience and self-reports with examples of using the application to communicate; its influence on knowledge, resistance to peer pressure, intention to communicate with people who matter, and use of condoms and test for STI and HIV; and condom use and STI and HIV testing. Outcomes were iteratively compared across transcripts, categorized, and synthesized into analytical summaries for interpretation.

#### Ethics Approval

The University of Zambia Biomedical Research Ethics Committee approved the study (institutional review board protocol number 811-2020), and the National Health Research Authority granted the authority to conduct the research.

## Results

### Enrollment

**Figure 2:**
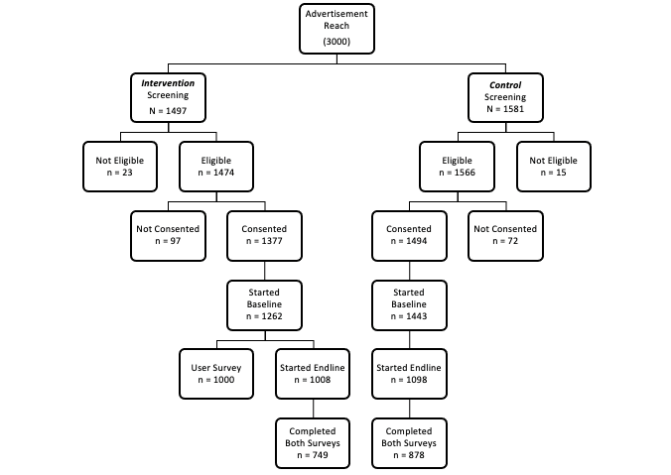
Participant flow.

The study targeted 3000 participants for the pilot study (Figure 2). Individuals who clicked on the recruitment advertisement were sent to a Qualtrics [30] webpage with a 4-question eligibility screener. We registered 3078 screener attempts, 98.77% (3040/3078) eligible records, 94.44% (2871/3040) consents, 94.44% (2705/2871) baselines of 16 questions (Multimedia Appendix 3), and 73.35% (2106/2871) endline surveys. The final sample comprised 1627 participants who completed both the baseline and endline surveys. We removed 230/1627 (14.1%) duplicates and ineligible accounts from the final data set. We observed a low attrition rate from screening to enrollment (2871/3078, 6.7%) and a reasonable completion rate (1627/2871, 56.7%).

### Participant Characteristics

Most of the 1627 individuals were single (1502/1613, 93.12%), with a median age of 22 (IQR 21-23) years and from averagely wealthy (868/1340, 64.78%) and respected (774/1293, 59.86%) families. Approximately half were men (822/1613, 50.96%), college educated (811/1615, 50.22%), and trainees or students (706/1614, 43.74%).

The comparison group had a higher proportion of college or university-trained individuals (462/872, 52.9%, vs 349/743, 46.9%; *P*=.002) and in the student or trainee category (424/872, 48.6%, vs 282/742, 38%; *P*<.001) than the intervention group (Multimedia Appendix 4).

The 2 groups were similar with regard to sexual health history, with 42.33% (682/1611) testing for HIV and 63.61% (1014/1594) testing for STIs (Multimedia Appendix 4, Table S3). The intervention group had a higher proportion of sexually active individuals (404/564, 76.0%, vs 396/521, 71.6%), who had sex in the past 6 months (294/510, 57.6%, vs 293/565, 51.9%), with slightly lower condom use (281/369, 76.2%, vs 317/402, 78.9%) and slightly more unplanned sex (194/369, 52.6%, vs 221/403, 54.8%) at the last sexual intercourse.

### Adjusted Average Treatment Effect for BITKZ Intervention

#### Overview

The intervention group was more likely to score higher on the intention to test for STI (aATE: 0.21; *P*=.01) and HIV (aATE: 0.32; *P*=.05) than the comparison group (Multimedia Appendix 4). The effect measures for resisting peer pressure increased more than 2-fold (*P*=.02), likely because of heterogeneity in responses and higher possible scores. No other statistically significant effects were observed.

The intervention group had a modest increase in the likelihood to score higher in SRH knowledge (aATE: 0.12), self-efficacy (aATE: 0.21), use of condoms at the next sexual intercourse (aATE: 0.19%), and confidence in getting an STI test (aATE: 0.18) and condoms (aATE: 0.29) when needed. The likelihood of scoring higher for permissive attitudes was 91% higher in the intervention group, although they were 8% less likely to score higher for sexual norms.

#### Intention to Test for STI and HIV and Use Condoms

Those aged 20 years had significantly higher adjusted odds of testing for STIs if symptomatic (adjusted odds ratio [aOR] 2.5, 95% CI 1.3-4.81) than those aged 18 years. Higher odds of HIV testing were observed among those aged 19 years (aOR 1.73, 95% CI 1.02-2.92), 20 years (aOR 1.7, 95% CI 1.01-2.86), and 23 years (aOR 1.81, 95% CI 1.14-2.91) years than among those aged 18 years. Those reporting average perceived wealth had lower odds of testing for STIs if symptomatic (aOR 0.69, 95% CI 0.52-0.93) and testing for HIV (aOR 0.68, 95% CI 0.54-0.86) than those identifying with above-average perceived wealth.

In the IDIs, more women than men expressed intent to test for STI and HIV, motivated by self-preservation and to secure their future. For example, a woman aged 22 years claimed the following:

To engage in sex, it has to be a safe one, so that I can continue with my education and my career.

Both men and women provided examples of taking up STI or HIV testing as they felt more knowledgeable, motivated, and self-confident, as encapsulated in the following quote:

We are, you know, ignorant about sex...before I used the application, I used to be frustrated that “Ok, I slept with that girl but ah!” I start asking questions “Maybe I get STIs and whatnot.” But once I started using the App I got motivated, I went for HIV testing, STIs like that, so that I am updated. Then issues of how to use a condom used to be a challenge. I didn’t know how, if I am about to have sex, how I am supposed to put on the condom or remove it [I: hmm] So when I started using the application, I know a lot. Yeah I know a lot. I became motivated and I even went for HIV testing.

Male, 22 years

Many participants felt nudged by the knowledge that “not all STIs have symptoms” and “that STIs can be treated. So, if at any time I find out that I have STIs, I don’t have to be worried...I can get help from a health clinic.” They thought that having such information helped those who “may say they feel stigmatized when they have that. But if they use this app, they will know their rights, and they’ll be able to learn and also to get tested for STIs whenever they have symptoms.”

#### Peer Pressure

The intervention group had a slightly higher mean score (19.81, 95% CI 19.56-20.06) than the comparison group (19.68, 95% CI 19.44-19.91) for the ability to resist peer pressure. In the IDIs, both men and women reported gaining insight into how peer pressure can lead to undesirable outcomes and reported having an internal debate to decide their course of action and learning strategies to resist peer pressure. Multimedia Appendix 5 contains illustrative quotes to show the effect of BITKZ on the ability to resist pressure, condom and contraceptive-related knowledge, and partner communication in BITKZ users’ own words.

### Condom Knowledge

At the endline, the level of condom-related knowledge was 35% higher (aOR 1.35, 95% CI 1.07-1.69) among those who received the intervention than among those in the control group (Multimedia Appendix 4). In addition, men had a 27% lower level of condom-related knowledge than women (aOR 0.73, 95% CI 0.58-0.92).

Conditional or matched logistic regression comparing baseline to endline by intervention status indicated significantly increased odds of improvement in knowledge regarding the need to try different types of condoms to suit both partners in both groups, more so among intervention (matched odds ratio 3.35, 95% CI 2.5-5.33) than comparison participants (matched odds ratio 2.03, 95% CI 1.34-3.08).

Ordinal regression on correct condom use indicated increased odds of scoring higher (0-6 possible) in knowledge on how to wear the condom among the intervention group (aOR 1.27, 95% CI 1.06-1.54) compared with the control group, among men (aOR 1.92, 95% CI 1.59-2.31) compared with women, and among those employed full time (aOR 1.67, 95% CI 1.06-2.63) compared with those reporting as unemployed (Multimedia Appendix 4).

In the interviews, young people expressed their happiness with the information about the different options for condoms that can be used during sexual intercourse available on the BITKZ application, as well as evidence of learning about contraceptive choices (Multimedia Appendix 5). All participants emphasized that an important lesson they had picked from the application visual guides was the correct way of using condoms:

We don’t have someone to tell us how to use condoms to take care of ourselves we are just doing things blindly.

### Communication With People Who Matter About SRH Topics

In descending order, participants felt they could most definitely seek advice from people who matter, defined as health care workers, boyfriends or girlfriends, friends, peers, other adults, teachers, siblings, parents, community leaders, and priests (Multimedia Appendix 4). Comparison group participants were more definite about their ability to seek advice or ideas on SRH from a priest (mean 2.4, SD 1.16 vs mean 2.52, SD 1.15; *P*=.04).

At baseline, 37.9% (318/838) of comparison group participants reported seeking advice from the church on SRH in the past month compared with 31.4% (227/724) among those in the intervention group (*P*=.001), a difference that persisted into the endline (260/741, 49.1%, vs 355/848, 35.1%; *P*=.05). At the endline, more comparison group than intervention group participants reported seeking SRH advice in the past month from family (502/844, 59.5%, vs 387/740, 52.3%; *P*=.02) and a professor or teacher (435/846, 51.4%, vs 335/742, 45.1%; *P*=.03).

Intervention group participants regarded talking to adults or parents about SRH as taboo because of moral censure, religious beliefs, sociocultural norms, and attitudes regarding adolescent sexual behavior. According to them, adults thought that “there are things adolescents should not know at that age”; otherwise “they may want to practice sex.” In addition, participants indicated that parents did not discuss condom use and cautioned them to “take it easy in life, there are a lot of diseases and do not misbehave.”

Young women in the intervention group disclosed that they felt generally *uncomfortable* and *shy* to have open conversations about sex. They felt less confident in their ability to discuss and insist on condom use with their partner, leaving them worried after having sex. Some participants explained that the application boosted their confidence in discussing safe sex with their partners. The relatable scenarios and discussion starters on the application provided guidance on how they could raise topics about condom use with sexual partners. Some women considered it important to have the application so that they could invite their partners to use it and open up discussions about safe sex (Multimedia Appendix 5).

Both young men and women reported that their friends commonly provided information that was often not complete or correct. All participants noted that the application created an environment for *smart knowledge* and *confidence* when discussing sex and SRH with friends. Many participants described recent efforts to share information gathered from the application with their friends and extended invitations to friends to access the application and act on its recommendations:

Yeah like I shared about the App, we read some stories and answered questions with about eight girls at my boarding house. So we later went to get condoms from the clinic, from our nearest clinic.

Female, 21 years

I had a conversation with one of my friends about using condoms [...], he said, “Now I can’t take action right now because those things [condoms] are too expensive!” That’s when I told him that, “My friend, these things, it’s not all about buying. You can just go to the clinic, you take.” That’s the action which he took. He went to the clinic and he was given those things.

Male, 23 years

### User Experience

The application captured linking data for 80.2% (601/749) of intervention participants, half of whom spent 17 (IQR 6-48) minutes on BITKZ. Among the intervention group, 85% (637/749) completed the user survey and rated BITKZ highly (excellent: 359/637, 56.4%; good: 237/637, 37.2% good). Most items under the constructs of aesthetic, engaging, functional, useful, and shareable were rated *very* by >60% of the respondents (Multimedia Appendix 4). Exceptions included the ability to personalize content (*very*; 302/637, 47.4%), likelihood of sharing if in own language (*very*; 360/637, 56.5%), vastness of content (*very*; 328/637, 51.5%), and willingness to pay (*very*; 205/637, 32.2%), although the proportion rating these items as *not at all* was <11%. IDI participants who rated the app *poor* explained that they had problems with functionality because of bandwidth issues.

## Discussion

### Principal Findings

The BITKZ application met its objective of increasing condom-related knowledge and resistance to peer pressure among young Zambians aged 18 to 24 years who use the internet. The BITKZ application did not achieve a statistically significant increase in communication about SRH, although BITKZ users described using it to inform and motivate people who matter to adopt healthier sexual choices. The intervention group had a higher intent to test for STIs and HIV, possibly as the application reduced stigma and fear associated with prolonged morbidity and infectiousness [41-44]. Although not statistically significant, possible modest improvements in sexual norms, self-efficacy, confidence to procure a condom, STI test, and intent to use condoms in the next sexual intercourse suggest that the BITKZ application may influence young adults to plan and practice safer sex [45,46]. Triggers and prompts that encourage site visits, use, and engagement among adolescents and young people need further investigation [47].

Our evaluation confirmed the need to include visual guides on correct condom use in comprehensive sexuality education offered in Zambia [48,49]. However, more research is needed on the effectiveness of educational materials using drawings of disembodied parts, particularly for communicating the correct use of female condoms [50]. The Zambian legal framework for the depiction of full images of men and women for sexual education needs clarification, given the prohibition of possessing *obscene* materials [51] and of transmitting them electronically [52]. This will require engagement and negotiation with the community and influential leaders; research ethics boards; regulatory bodies; the Zambia Information, Communication, and Technology Authority; and the Ministry of Justice. These and other materials can be further culturally adapted and produced remotely at low cost and at scale, as noted by the Kenyan Tumaini project, which developed and piloted a smartphone game intervention to improve SRH among young people [23].

Increased resistance to peer pressure may have been easier in our older study population than in preteens and early teenagers, as targeted by other studies [23,47]. The Tumaini project [23] addressed but did not measure the effect of peer pressure on those aged 11 to 14 years in Kenya. A digital storytelling intervention conducted in South Africa [47] found that exposure to multiple alternate views led high school girls to better understand their own emotions and behavior, including with regard to peer pressure. In our study, scenarios resonated with BITKZ users, leading to introspection and the intention to resist peer pressure. BITKZ use data, including user interactions, will be further examined for evidence of unintended peer pressure on the BITKZ platform [53].

Closson et al [54] and other comprehensive reviews [55] illustrate the correlation of personal experience and norms with those of peers, as well as the differential effects of sexual norms and behavior change interventions by gender in SSA. Unlike the previous review, our intervention reflected normative changes, possibly because of resistance to peer pressure and improved communication with peers and sexual partners [54]. However, young men in our intervention group learned more about correct condom use than did women, suggesting gendered interests and loci of control [54]. We will further analyze our data, disaggregated by sex and age groups, to understand the differential effect of this intervention by drawing on the application log files to capture use and estimate dose-response.

In addition, higher sexual permissiveness but lower sexual norm scores may reflect a healthier, sex-positive attitude, which was found to be safer for STI prevention in the United States [43]. Despite these encouraging results on condom use knowledge and intention to test for STI and HIV, gender relations and lack of youth-centered approaches at clinics may bar the uptake of STI and HIV prevention and care-seeking behaviors. These social and structural barriers have impeded STI prevention in SSA [56-60]. Digital health innovations for training SRH care providers can link adolescents and young people to needed services and allow for the social monitoring of SRH services for adolescents and young people [61,62].

Having accurate knowledge of STIs can reduce stigma and increase intention to test for STIs [41-44] and sexual confidence [63]. Although our study demonstrated increased intention, the intervention was insufficient to open conversations between adults and adolescents and young people, which may make it less likely that adolescents and young people will access STI testing [63]. Modernization in the timing and content of *sexual teachings* [64,65] and health programs that increase adolescents’ and young peoples’ assertiveness can increase SRH communication with parents and other significant adults, with anticipated benefits [63]. In addition, parent-based interventions show a significant association with improved condom use and parent-child sexual communication, especially when focused on young adolescents and targeted at both parents and adolescents [66]. Evidence-based choice and design of a parent-child intervention and cocreation processes would require intergenerational engagement and mediation to diminish parents’ discomfort and fears about communicating with their children on sexual matters [8,23,67-69]. Intergenerational games [23,70] and digital storytelling can remove some of the barriers to parent-child communication.

### Limitations

Owing to the COVID-19 pandemic restrictions, we conducted web-based recruitment, data collection, and BITKZ implementation. As a result, our sample may be more educated, technology savvy, urban, male, and wealthy than the general adolescents and young people population [71]. In addition, the COVID-19 pandemic determined our exclusively web-based BITKZ design. A more inclusive combined web-based and in-person design may deepen engagement and help identify misconceptions and adolescents and young people–specific language [9,72]. Although use data indicate good engagement, an analysis of proximity to the time of the survey, time on the application, and time on each feature is needed. Although aligned with our intention-to-treat analysis, including exposure status can help estimate the efficacy of our intervention and its relevance based on demographic characteristics.

Web-based data collection did not allow us to confirm age and residence, although all provided Zambian phone numbers to receive communication and reimbursement in ZMW and were required to complete all the screening questions so that they would not learn why they were considered ineligible. Being completely on the web, we limited the number of survey questions and could not validate the truncated scales for the theory of planned behavior constructs [34]. We did not randomize enrollees to the intervention or comparison groups, although both groups did not differ in outcome variables at baseline. Endline improvements in the comparison group suggested the influence of maturation and testing effect [73]. This, together with the high baseline scores for both groups, may have limited the power to detect small but significant changes because of BITKZ.

We cannot rule out courtesy, recall, and social desirability bias among participants responding to IDIs [73]. Owing to time, financial, and COVID-19 restrictions, we did not measure self-reported behavior changes validated by biomarkers. A longer follow-up time is required to estimate continued engagement with the application and its medium- to long-term impact on attitude, norms, intention, and behavior. In addition, comparison group enrollment was conducted in series rather than in parallel with the intervention group; however, we anticipate selection bias to be minimal and biased toward the null, given a brief enrollment period and order of enrollment (intervention and then comparison group).

### Comparison With Prior Work

We found no comparable study on similar DHIs for heterosexual young people either because of differences in targeted age (<18 years) or setting (school based) in SSA [23,74,75]. Similar programs targeting young people aged 18 to 24 years come from the clinic and web-based settings in the United States [76] and university settings in the Netherlands [77]. In addition, the Dutch *Justify Your Love* study targeted heterosexual couples in a new relationship [77]. All 5 DHIs discussed herein used random assignment of participants as control and 1 to 2 intervention conditions to provide information, motivation, and problem-solving and behavioral skills in English. In addition, BITKZ, Justify Your Love [77], and the Tumaini pilot in Kenya [23] addressed communication skills.

The DHIs differed in form and content, informed by context, formative research, behavior change theories, educational tools, and cocreation or wide consultation. Similar to BITKZ, the 2 trials were persona based [23,75] and had reward features [23,75,77]. Unlike BITKZ, 3 were set in virtual worlds [23,75,77]: one designed for offline users [75] and another for Android smartphone [23] users. The 3 DHIs in SSA stipulated duration, venues, and linear flow, some with material or teaching support [23,74,75]. The CyberSenga trial in Uganda was unique in its use of adult traditional figures as role models and completion certifications [74]. Non-SSA participants received up to €20 (US $20.86) for full participation in cash [77] or gift certificates [76], whereas BITKZ participants received up to €4 (US $4.17) in incentives, which may explain the relatively modest attrition.

Differences in purpose and design choices led to differences in the reported measures. Games and gamification have been proven to improve knowledge, motivation, and engagement in learning about SRH [74], goal setting and risk avoidance [23,74], and SRH knowledge [23,74]. Unlike the non-SSA trials YouthNet [76] and Justify Your Love [77], SSA-based DHIs reported a statistically significant increase in SRH self-efficacy [23] and, although statistically nonsignificant, increased the likelihood to remain abstinent [74]. CyberSenga [78] and Justify Your Love [77] reported increased condom use unlike YouthNet [76]. The Justify Your Love intervention did not change attitude, normative beliefs, skills toward maintenance of condom use or STI testing, and the uptake of STI testing [77]. YouthNet reported no differences in awareness of HIV or sexually transmitted disease risk and attitudes toward condom use [76].

### Conclusions

This evaluation study successfully used social media to recruit adolescents and young people aged 18 to 24 years to participate in an exclusively web-based SRH program. Young Zambians with internet access have a high awareness of SRH issues. BITKZ provided modest gains in intention to test for STIs, possibly because of the novelty of this concept vis-à-vis HIV, and in correct condom use because of insufficient prior knowledge.

## Appendix

Multimedia Appendix 1 Be in the know Zambia objectives and theoretical basis.

Multimedia Appendix 2 Be in the know Zambia application features.

Multimedia Appendix 3 Be in the know Zambia survey instrument.

Multimedia Appendix 4 Quantitative tables.

Multimedia Appendix 5 Supporting quotes.

## Abbreviations

aATE: adjusted average treatment effect
aOR: adjusted odds ratio
BITKZ: Be in the know Zambia
DHI: digital health intervention
IDI: in-depth interview
SRH: sexual and reproductive health
SSA: sub-Saharan Africa
STI: sexually transmitted infection
ZMW: Zambian Kwacha

## References

[1] Khalifa A, Stover J, Mahy M, Idele P, Porth T, Lwamba C (2019). Demographic change and HIV epidemic projections to 2050 for adolescents and young people aged 15-24. Glob Health Action.

[2] UN Joint Programme on HIV/AIDS (2014). Fast-track: commitments to end AIDS by 2030. UNAIDS.

[3] Risher KA, Cori A, Reniers G, Marston M, Calvert C, Crampin A, Dadirai T, Dube A, Gregson S, Herbst K, Lutalo T, Moorhouse L, Mtenga B, Nabukalu D, Newton R, Price AJ, Tlhajoane M, Todd J, Tomlin K, Urassa M, Vandormael A, Fraser C, Slaymaker E, Eaton JW, ALPHA Network (2021). Age patterns of HIV incidence in eastern and southern Africa: a modelling analysis of observational population-based cohort studies. Lancet HIV.

[4] Birdthistle I, Tanton C, Tomita A, de Graaf K, Schaffnit S, Tanser F, Slaymaker E (2019). Recent levels and trends in HIV incidence rates among adolescent girls and young women in ten high-prevalence African countries: a systematic review and meta-analysis. Lancet Glob Health.

[5] Roberts L (2021). How COVID is derailing the fight against HIV, TB and malaria. Nature.

[6] Brown LB, Spinelli MA, Gandhi M (2021). The interplay between HIV and COVID-19: summary of the data and responses to date. Curr Opin HIV AIDS.

[7] Wadham E, Green C, Debattista J, Somerset S, Sav A (2019). New digital media interventions for sexual health promotion among young people: a systematic review. Sex Health.

[8] Daher J, Vijh R, Linthwaite B, Dave S, Kim J, Dheda K, Peter T, Pai NP (2017). Do digital innovations for HIV and sexually transmitted infections work? Results from a systematic review (1996-2017). BMJ Open.

[9] Manby L, Aicken C, Delgrange M, Bailey JV (2022). Effectiveness of eHealth interventions for HIV prevention and management in Sub-Saharan Africa: systematic review and meta-analyses. AIDS Behav.

[10] Bailey JV, Wayal S, Aicken CR, Webster R, Mercer CH, Nazareth I, Rait G, Peacock R, Murray E (2021). Interactive digital interventions for prevention of sexually transmitted HIV. AIDS.

[11] Tarnate PS, Serrano KR, Torres-Ticzon VM, Senen KA (2019). Effectiveness of digital media technologybased interventions on HIV & STI risk reduction among young people: a metaanalysis. Pediatr Infect Dis Soc Philippines J.

[12] (2017). Towards a global HIV prevention coalition and road map. UNAIDS.

[13] Nakazwe C, Michelo C, Sandøy IF, Fylkesnes K (2019). Contrasting HIV prevalence trends among young women and men in Zambia in the past 12 years: data from demographic and health surveys 2002-2014. BMC Infect Dis.

[14] Shanaube K, Macleod D, Chaila MJ, Mackworth-Young C, Hoddinott G, Schaap A, Floyd S, Bock P, Hayes R, Fidler S, Ayles H (2021). HIV care cascade among adolescents in a "test and treat" community-based intervention: HPTN 071 (PopART) for youth study. J Adolesc Health.

[15] (2020). Country Progress Report - Zambia: Global AIDS Monitoring 2020. Joint United Nations Programme on HIV/AIDS.

[16] (2017). The safeguard young people programme: three years on: addressing the urgent needs of youth across Southern Africa. United Nations Population Fund.

[17] (2019). End of project evaluation of the BBC media action radio waves and Tikambe projects in Zambia: final report. Sida Decentralised Evaluation.

[18] U-Report Zambia. United Nations International Children's Emergency Fund.

[19] Ministry of Health, Zambia (2020). COVID-19 update #12 summary. Facebook.

[20] Nolan C, Packel L, Hope R, Levine J, Baringer L, Gatare E, Umubyeyi A, Sayinzoga F, Mugisha M, Turatsinze J, Naganza A, Idelson L, Bertozzi S, McCoy S (2020). Design and impact evaluation of a digital reproductive health program in Rwanda using a cluster randomized design: study protocol. BMC Public Health.

[21] Castle S, Martha S (2019). Family planning and youth in West Africa: mass media, digital media, and social and behavior change communication strategies. Breakthrough RESEARCH Literature Review.

[22] Faust L, Yaya S (2018). The effect of HIV educational interventions on HIV-related knowledge, condom use, and HIV incidence in sub-Saharan Africa: a systematic review and meta-analysis. BMC Public Health.

[23] Winskell K, Sabben G, Akelo V, Ondeng'e K, Obong'o C, Stephenson R, Warhol D, Mudhune V (2018). A smartphone game-based intervention (Tumaini) to prevent HIV among young Africans: pilot randomized controlled trial. JMIR Mhealth Uhealth.

[24] (2018). Qualtrics.

[25] Kok G, Gottlieb NH, Peters GJ, Mullen PD, Parcel GS, Ruiter RA, Fernández ME, Markham C, Bartholomew LK (2016). A taxonomy of behaviour change methods: an intervention mapping approach. Health Psychol Rev.

[26] Turner JC, Ellemers N, Spears R, Doosje B (1999). Social identity theory: where are we now?. Social Identity: Context, Commitment, Content.

[27] Protogerou C, Johnson BT, Hagger MS (2018). An integrated model of condom use in Sub-Saharan African youth: a meta-analysis. Health Psychol.

[28] Klasnja P, Hekler EB (2018). Rethinking evaluations of mHealth systems for behavior change. GetMobile.

[29] DeSmet A, Shegog R, Van Ryckeghem D, Crombez G, De Bourdeaudhuij I (2015). A systematic review and meta-analysis of interventions for sexual health promotion involving serious digital games. Games Health J.

[30] Jessen S, Mirkovic J, Ruland CM (2018). Creating gameful design in mHealth: a participatory co-design approach. JMIR Mhealth Uhealth.

[31] (2012). cGrate.

[32] Stanton B, Deveaux L, Lunn S, Yu S, Brathwaite N, Li X, Cottrell L, Harris C, Clemens R, Marshall S (2009). Condom-use skills checklist: a proxy for assessing condom-use knowledge and skills when direct observation is not possible. J Health Popul Nutr.

[33] Creswell JW (2010). Educational Research: Planning, Conducting, and Evaluating Quantitative and Qualitative Research. 4th edition.

[34] Ajzen I (1991). The theory of planned behavior. Organ Behav Hum Decis Process.

[35] Muhammad NA, Shamsuddin K, Mohd Amin R, Omar K, Thurasamy R (2017). Questionnaire development and validity to measure sexual intention among youth in Malaysia. BMC Public Health.

[36] (2013). National Survey of Adolescents and Young Adults: Sexual health knowledge, attitudes and experiences. Kaiser Family Foundation.

[37] Lewis IM, Watson B, White KM (2010). Response efficacy: the key to minimizing rejection and maximizing acceptance of emotion-based anti-speeding messages. Accid Anal Prev.

[38] (2019). Stata – Statistical Software: Release 16.1. StatCorp.

[39] Wickham H, Francois R, Henry L, Müller K (2021). dplyr: A Grammar of Data Manipulation. R package version 1.0.7.

[40] Watkins DC (2017). Rapid and rigorous qualitative data analysis: the “RADaR” technique for applied research. Int J Qual Methods.

[41] Garcia PJ, Miranda AE, Gupta S, Garland SM, Escobar ME, Fortenberry JD, International Union Against Sexually Transmitted Infections (2021). The role of sexually transmitted infections (STI) prevention and control programs in reducing gender, sexual and STI-related stigma. EClinicalMedicine.

[42] Lee AS, Cody SL (2020). The stigma of sexually transmitted infections. Nurs Clin North Am.

[43] Thomas JA, Ditchman N, Beedle RB (2020). The impact of knowledge, self-efficacy, and stigma on STI testing intention among college students. J Am Coll Health (forthcoming).

[44] Bender S, Hill K, Information Resources Management Association (USA) (2021). Sexually transmitted infections – diagnoses, stigma, and mental health. Research Anthology on Mental Health Stigma, Education, and Treatment.

[45] Darabi F, Yaseri M, Kaveh MH, Khalajabadi Farahani F, Majlessi F, Shojaeizadeh D (2017). The effect of a theory of planned behavior-based educational intervention on sexual and reproductive health in Iranian adolescent girls: a randomized controlled trial. J Res Health Sci.

[46] Morales A, Espada JP, Orgilés M, Escribano S, Johnson BT, Lightfoot M (2018). Interventions to reduce risk for sexually transmitted infections in adolescents: a meta-analysis of trials, 2008-2016. PLoS One.

[47] Quilling R, Pieterse G (2018). To see someone else’s perspective: a case for digital stories in schools. J Indep Teach Learn.

[48] Barrett M, Laris BA, Anderson P, Baumler E, Gerber A, Kesler K, Coyle K (2021). Condom use and error experience among young adolescents: implications for classroom instruction. Health Promot Pract.

[49] Levkoff L, Kempner M (2021). We’re teaching about condoms all wrong: how sex educators reinforce negative attitudes and misinformation about condoms and how to change that. Am J Sex Educ.

[50] Chatterjee K (2018). What can we learn about the female condom online? An analysis of visual representations of the female condom on the Internet. Atl J Commun.

[51] Penal Code Section 177(1) Obscene matter or things in Chapter 87 of the Laws of Zambia. Laws of Zambia, Republic of Zambia.

[52] Section 102 of the Electronic Communications and Transactions ACT of Zambia. United Nations Office on Drugs and Crime.

[53] Goodyear V, Armour K, Wood H (2018). The Impact of Social Media on Young People’s Health and Wellbeing: Evidence, Guidelines and Actions. University of Birmingham.

[54] Closson K, Dietrich JJ, Lachowsky NJ, Nkala B, Palmer A, Cui Z, Beksinska M, Smit JA, Hogg RS, Gray G, Miller CL, Kaida A (2018). Sexual self-efficacy and gender: a review of condom use and sexual negotiation among young men and women in Sub-Saharan Africa. J Sex Res.

[55] Cislaghi B, Shakya H (2018). Social norms and adolescents' sexual health: an introduction for practitioners working in low and mid-income African countries. Afr J Reprod Health.

[56] Wood JM, Harries J, Kalichman M, Kalichman S, Nkoko K, Mathews C (2018). Exploring motivation to notify and barriers to partner notification of sexually transmitted infections in South Africa: a qualitative study. BMC Public Health.

[57] Nyblade L, Stockton M, Nyato D, Wamoyi J (2017). Perceived, anticipated and experienced stigma: exploring manifestations and implications for young people's sexual and reproductive health and access to care in North-Western Tanzania. Cult Health Sex.

[58] Chitneni P, Beksinska M, Dietrich JJ, Jaggernath M, Closson K, Smith P, Lewis DA, Matthews LT, Smit J, Ndung'u T, Brockman M, Gray G, Kaida A, AYAZAZI Research Team (2020). Partner notification and treatment outcomes among South African adolescents and young adults diagnosed with a sexually transmitted infection via laboratory-based screening. Int J STD AIDS.

[59] Nmadu AG, Mohamed S, Usman NO (2020). Adolescents’ utilization of reproductive health services in Kaduna, Nigeria: the role of stigma. Vulnerable Child Youth Stud.

[60] Bylund S, Målqvist M, Peter N, Herzig van Wees S (2020). Negotiating social norms, the legacy of vertical health initiatives and contradicting health policies: a qualitative study of health professionals' perceptions and attitudes of providing adolescent sexual and reproductive health care in Arusha and Kilimanjaro region, Tanzania. Glob Health Action.

[61] Hager B, Hasselberg M, Arzubi E, Betlinski J, Duncan M, Richman J, Raney LE (2018). Leveraging behavioral health expertise: practices and potential of the project ECHO approach to virtually integrating care in underserved areas. Psychiatr Serv.

[62] Manlove J, Whitfield B, Finocharo J, Cook E (2021). Lessons learned from replicating a randomized control trial evaluation of an app-based sexual health program. Int J Environ Res Public Health.

[63] Scheinfeld E (2021). Shame and STIs: an exploration of emerging adult students' felt shame and stigma towards getting tested for and disclosing sexually transmitted infections. Int J Environ Res Public Health.

[64] Fumpa-Makano R (2019). Girls’ initiation ceremonies in Zambia: reflections on their role in girl child educational advancement. Int J Arts Soc Sci.

[65] Kok MC, van Eldik Z, Kakal T, Munthali A, Menon JA, Pires P, Baatsen P, van der Kwaak A (2022). Being dragged into adulthood? Young people's agency concerning sex, relationships and marriage in Malawi, Mozambique and Zambia. Cult Health Sex.

[66] Widman L, Evans R, Javidi H, Choukas-Bradley S (2019). Assessment of parent-based interventions for adolescent sexual health: a systematic review and meta-analysis. JAMA Pediatr.

[67] Wamoyi J, Fenwick A, Urassa M, Zaba B, Stones W (2010). Parent-child communication about sexual and reproductive health in rural Tanzania: implications for young people's sexual health interventions. Reprod Health.

[68] Isaksen KJ, Musonda P, Sandøy IF (2020). Parent-child communication about sexual issues in Zambia: a cross sectional study of adolescent girls and their parents. BMC Public Health.

[69] Nyimbili F, Mainza R, Mumba L, Katunansa B (2019). Teacher and parental involvement in providing comprehensive sexuality education in selected primary schools of Kalomo district of Zambia. J Adult Educ.

[70] D'Cruz J, Santa Maria D, Dube S, Markham C, McLaughlin J, Wilkerson JM, Peskin MF, Tortolero S, Shegog R (2015). Promoting parent-child sexual health dialogue with an intergenerational game: parent and youth perspectives. Games Health J.

[71] Kemp S (2021). Digital 2021: Zambia. Datareportal.

[72] Simungala G, Jimaima H (2020). Multilingual realities of language contact at the University of Zambia. J Asian Afr Stud.

[73] Barnes BR, Laher S, Fynn A, Kramer S (2019). Quasi-experimental designs in applied behavioural health research. Transforming Research Methods in the Social Sciences: Case Studies from South Africa.

[74] Ybarra ML, Korchmaros JD, Prescott TL, Birungi R (2015). A randomized controlled trial to increase HIV preventive information, motivation, and behavioral skills in Ugandan adolescents. Ann Behav Med.

[75] Haruna H, Hu X, Chu SK, Mellecker RR, Gabriel G, Ndekao PS (2018). Improving sexual health education programs for adolescent students through game-based learning and gamification. Int J Environ Res Public Health.

[76] Bull S, Pratte K, Whitesell N, Rietmeijer C, McFarlane M (2009). Effects of an Internet-based intervention for HIV prevention: the Youthnet trials. AIDS Behav.

[77] Mevissen FE, Ruiter RA, Meertens RM, Zimbile F, Schaalma HP (2011). Justify your love: testing an online STI-risk communication intervention designed to promote condom use and STI-testing. Psychol Health.

[78] Ybarra ML, Bull SS, Prescott TL, Korchmaros JD, Bangsberg DR, Kiwanuka JP (2013). Adolescent abstinence and unprotected sex in CyberSenga, an Internet-based HIV prevention program: randomized clinical trial of efficacy. PLoS One.

