# Supplementary material for: Pilot Implementation of a User-Driven, Web-Based Application Designed to Improve Sexual Health Knowledge and Communication Among Young Zambians: Mixed Methods Study
Source: J Med Internet Res. 2022 Jul 7;24(7):e37600. doi: 10.2196/37600 (PMC9305403; doi:10.2196/37600)
Supplement: Multimedia Appendix 1 [file jmir_v24i7e37600_app1.docx]

Supplement 1: BITKZ Objectives and Theoretical Basis

| Based on 18 IDIs conducted between 13 August 2020 – 5 October 2020, as well as co-creation work from 1 October 2020 – 19 November 2020, we developed an e-health intervention *Be in the Know Zambia* as follows:  **Identification of unsafe sexual behaviours and underlying cause**:   1. Young people reported pain when using condoms indicating the need to know about types and correct use of condoms. 2. Sexual and gender norms promote unplanned sex:    1. Young men feel pressured and teased into having sex by peers    2. Young women feel obliged to have sex when they have accepted food and drink for their friends and self. 3. Young people prefer not to use condoms with regular partners to signal trust, as more pleasurable and fear of losing their intimate relationship. 4. Parents threaten young people with dire consequences for unplanned pregnancies but do not explain how to prevent them.   **Identification of pathways of change**:   1. Young people know about types and correct use of condoms. 2. Young people learn of and from others like them in similar situations who make healthy choices and are able to resist peer pressure 3. Young people are able to talk to people who matter to them about things that are important to them   **Defining intervention objectives**:   1. Young people have access to accurate, easily understood information on condoms 2. Young people have access to accurate information and emotional support to prevent unplanned pregnancies and STIs 3. Young people are able to open up conversations with people who matter to them   **Application of behaviour change methods**:  Table A presents the methods chosen, their definitions and application to increase knowledge, emotional support, and communication that promote changes in sexual attitudes, norms and intentions as adapted from Kok et al [25].  TABLE A: Application of Behaviour Change Methods to Achieve Intervention Objectives   \| **Method** \| **Definition** \| **Operationalization** \| \| --- \| --- \| --- \| \| **Tailoring**  **Using imagery**  **Cultural similarity** \| Matching the intervention or components to previously measured characteristics of the participant.  Using artifacts that have a similar appearance to some subject.  Using characteristics of the target group in source, message, and channel. \| ***Be in the Know Zambia – Application***  User chooses character who they identify with and follows them through a scenario (**comic strip**).  **Characters, settings, information & framing**: Selected based on IDIs and co-creation processes  **FAQs/Top-tips**: User guides themselves through other content exploring the information they are most interested in / is most relevant to them.  **Comic strips/Lets talk cards**: They are able to discuss/share through the app. \| \| **Framing** \| Using gain or loss -framed messages on advantages/not of performing/not the healthy behavior \| **Top-tips, visual guides:** Gain-framed messages emphasizing the advantages of performing the healthy behavior \| \| **Verbal persuasion** \| Messages that suggest participant possesses certain capabilities. \| **Top-tips:** Positive messages throughout \| \| **Scenario-based risk information** \| Information to aid image construction of the ways in which a future loss or accident might occur. \| **Comic strips** Character dilemmas highlight possible risks in common scenarios \| \| **Mobilizing social support & networks** \| Prompting SBC in order to provide instrumental and emotional, appraisal and social support. \| **Lets talk cards:** Designed to be easily shared over social media to peer networks to provide information and spark discussion. Encouraging self- reflection, peer and partner discussions \| \| **Active learning**  **Discussion**  **Positive reinforcement** \| Encouraging learning from goal-driven activities and  consideration of a topic in open informal debate. \| **Quizzes** with final scores  **Comic strips** asking for comments on character dilemmas for empathy and interpretation  **Let's talk cards** encouraging self-reflection & peer and partner discussions collecting 'badges' for completing certain tasks \| \| **Implementation intentions** \| Prompting making if-then plans that link situational cues with responses that are effective in attaining goals or desired outcomes. \| **Comic strips** Character dilemmas are framed as common issues that users may need to overcome - user asked to give advice and make their own plans if they face a similar dilemma. \| \| **Goal setting** \| Prompting planning what the person will do, including a definition of goal-directed behaviors that result in the target behavior. \| **Let's talk cards** encourage self-reflection including thinking through user goals and plans for how these might be achieved. Support open discussion of goals with partners and peers. \| |
| --- | --- | --- | --- | --- | --- | --- | --- | --- | --- | --- | --- | --- | --- | --- | --- | --- | --- | --- | --- | --- | --- | --- | --- | --- | --- | --- | --- |
