# Supplementary material for: Pilot Implementation of a User-Driven, Web-Based Application Designed to Improve Sexual Health Knowledge and Communication Among Young Zambians: Mixed Methods Study
Source: J Med Internet Res. 2022 Jul 7;24(7):e37600. doi: 10.2196/37600 (PMC9305403; doi:10.2196/37600)
Supplement: Multimedia Appendix 2 [file jmir_v24i7e37600_app2.pptx]

## Slide 1
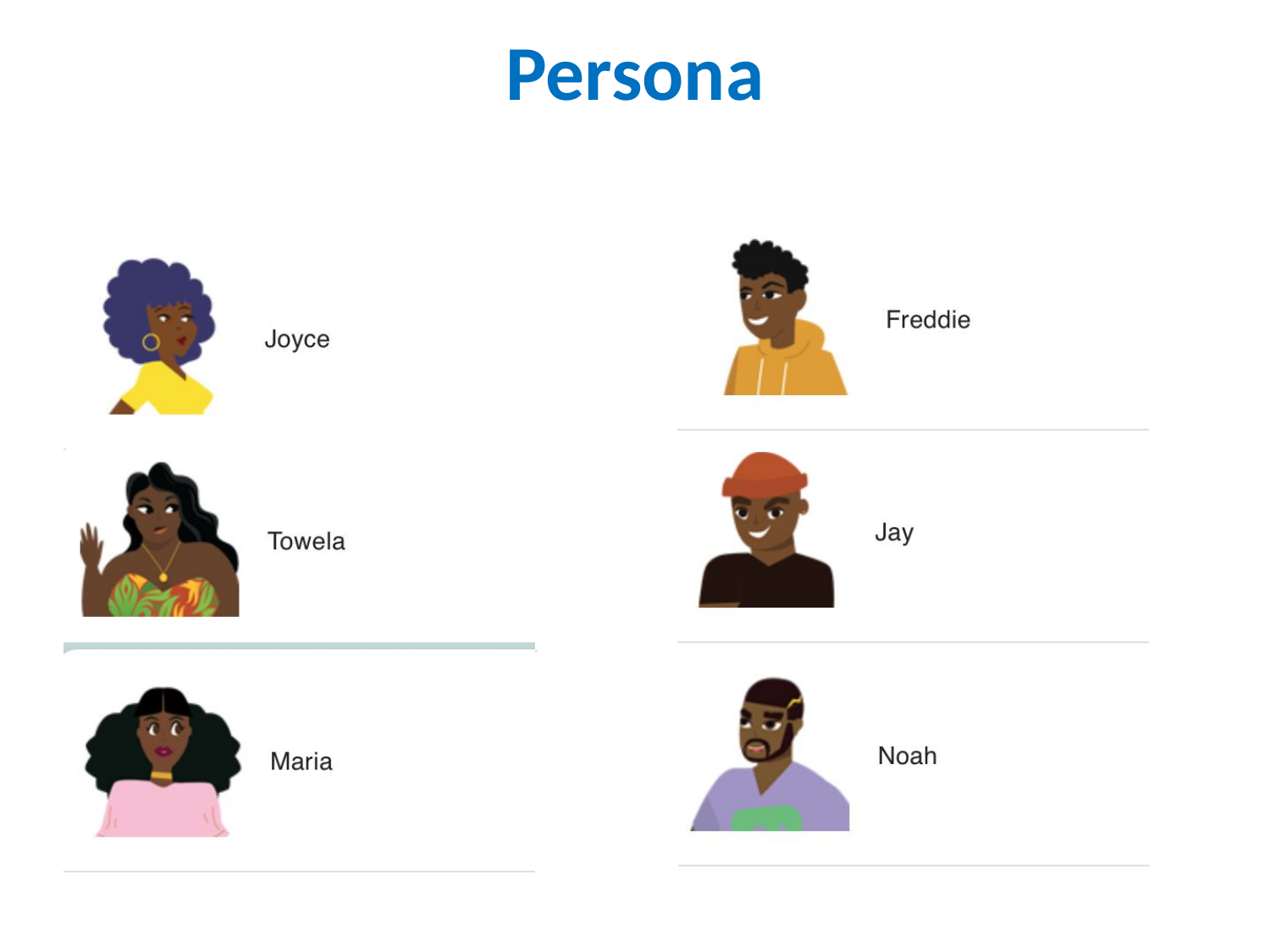

# Persona

## Slide 2
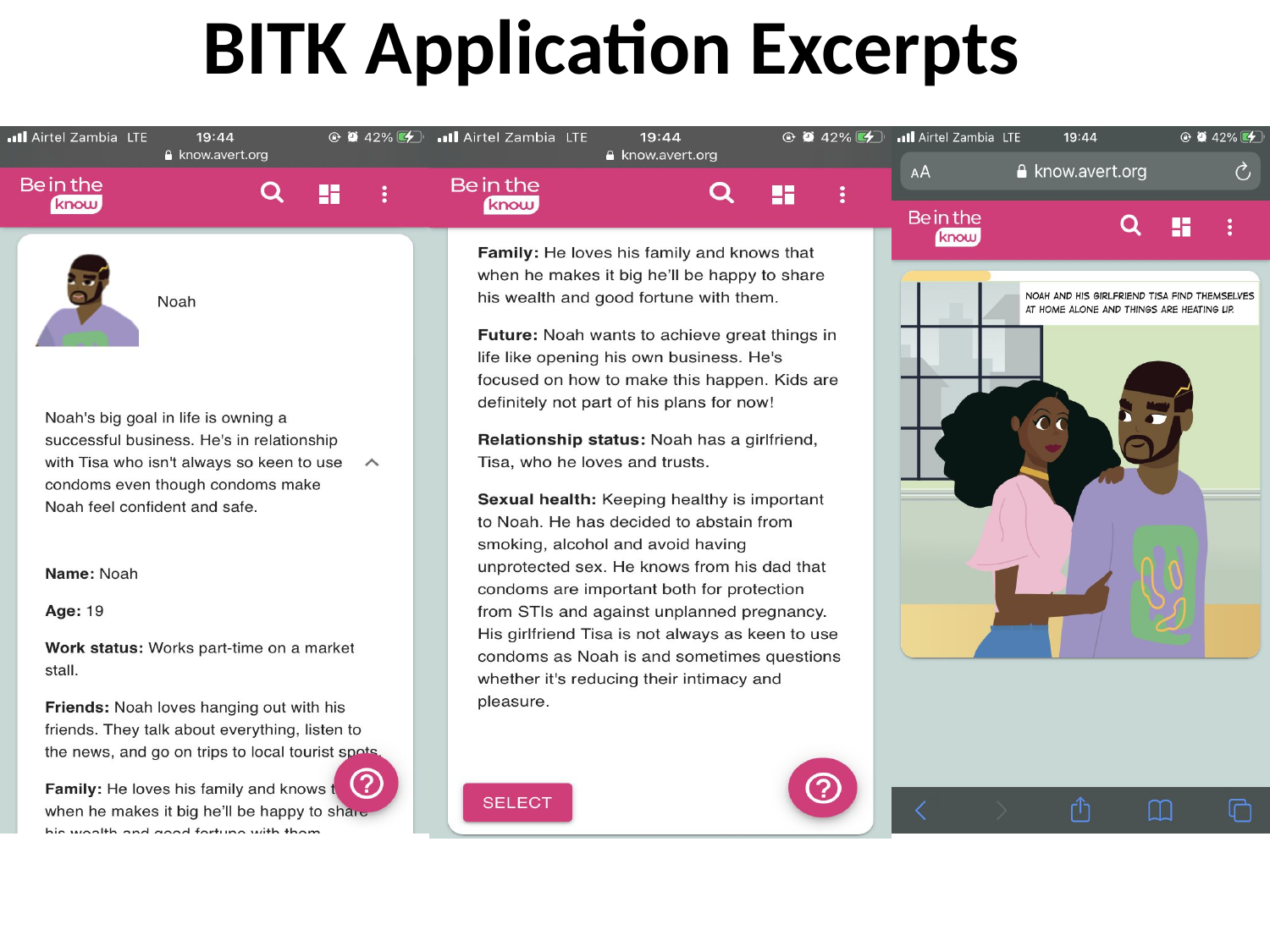

# BITK Application Excerpts

## Slide 3
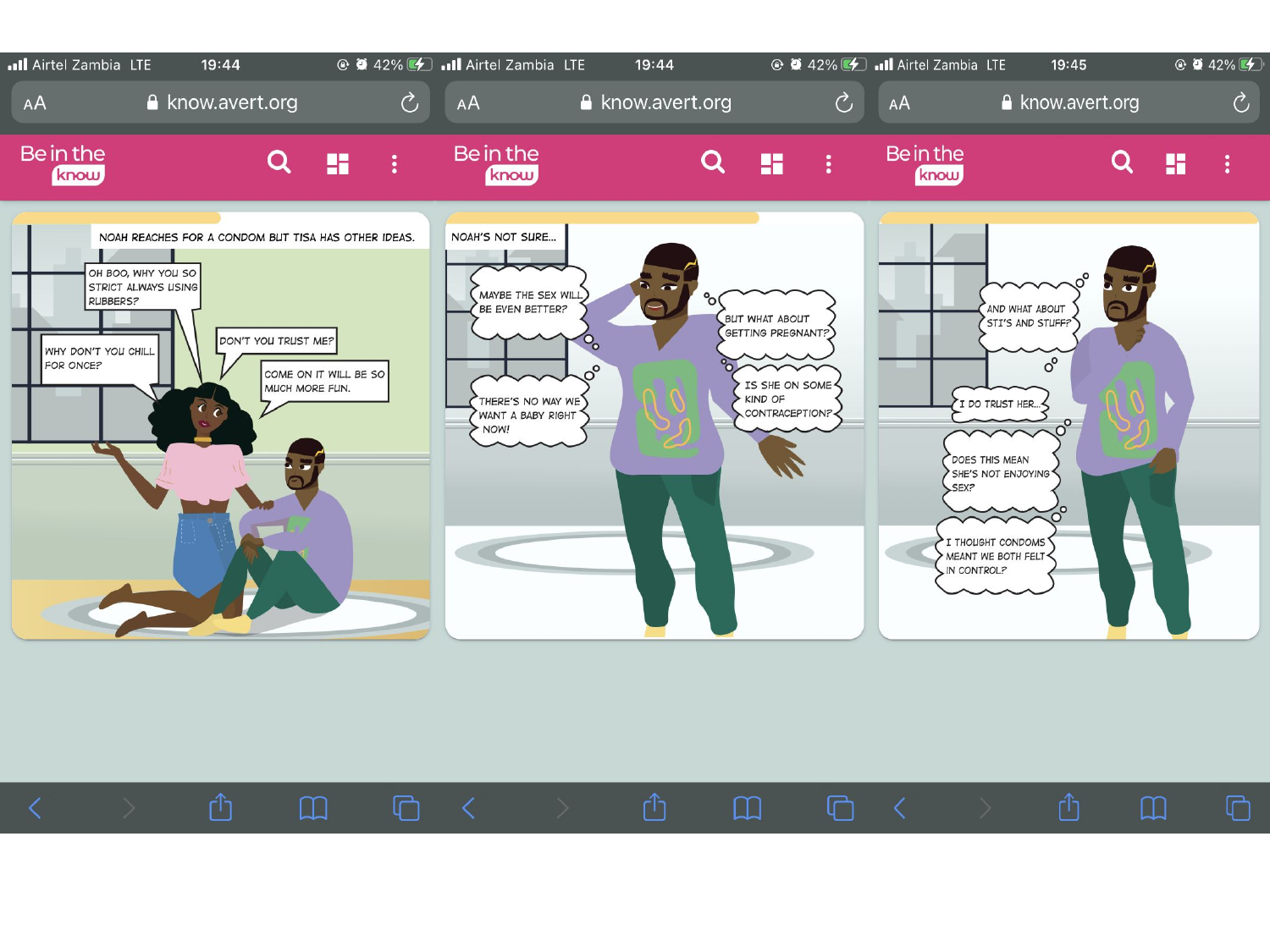

## Slide 4
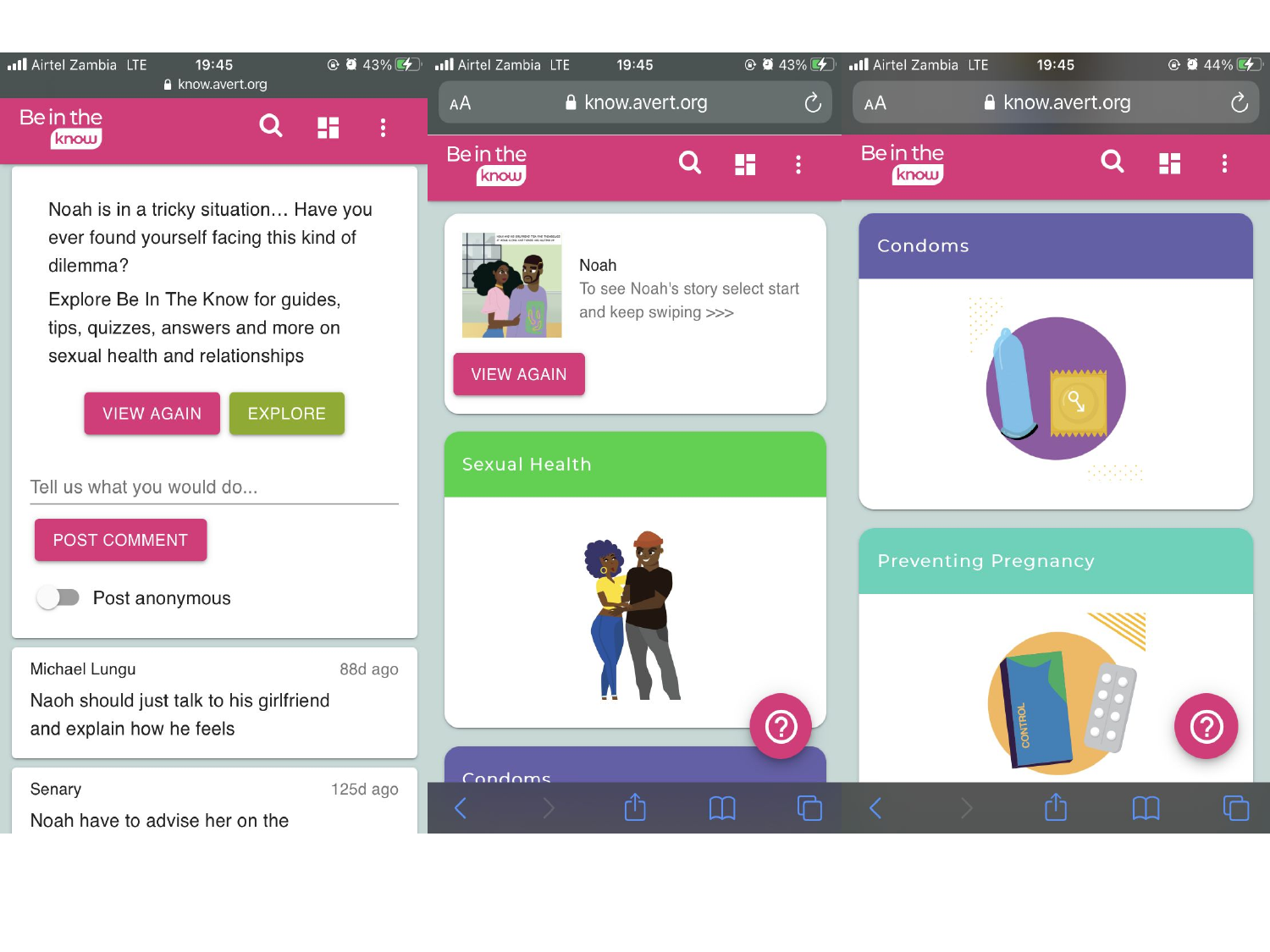

## Slide 5
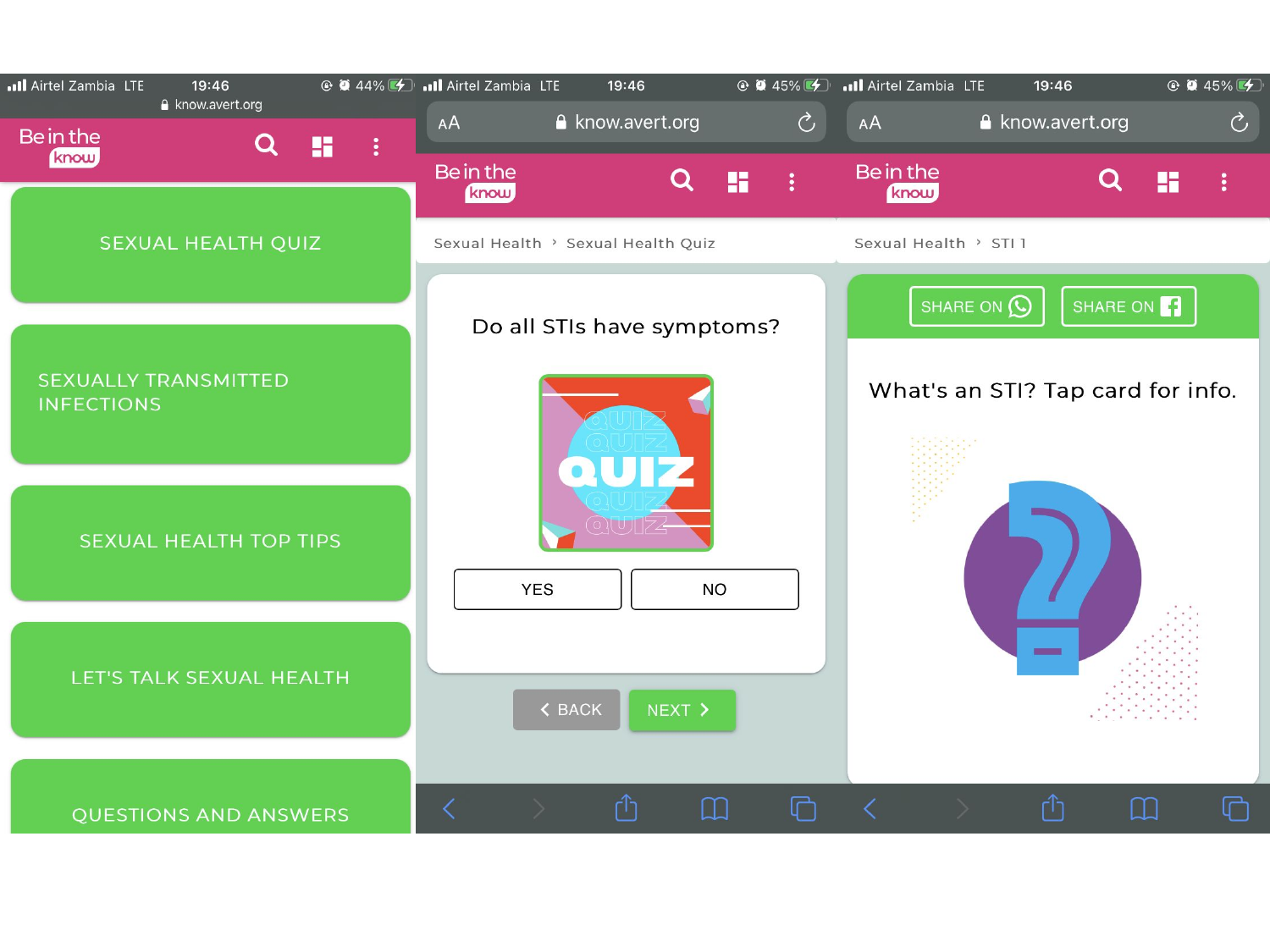

## Slide 6
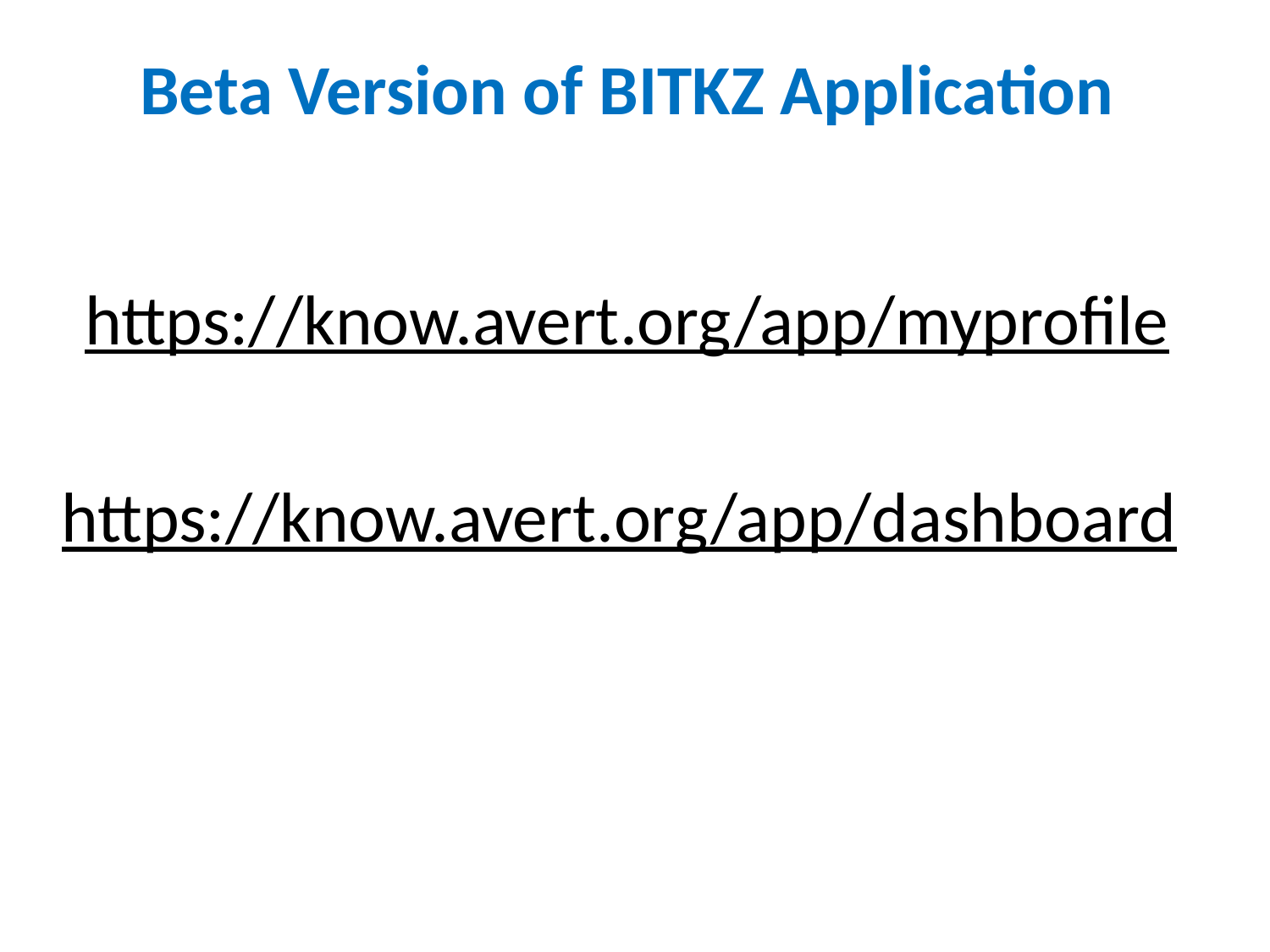

# Beta Version of BITKZ Application
https://know.avert.org/app/myprofile
https://know.avert.org/app/dashboard
