# Supplementary material for: Pilot Implementation of a User-Driven, Web-Based Application Designed to Improve Sexual Health Knowledge and Communication Among Young Zambians: Mixed Methods Study
Source: J Med Internet Res. 2022 Jul 7;24(7):e37600. doi: 10.2196/37600 (PMC9305403; doi:10.2196/37600)
Supplement: Multimedia Appendix 3 [file jmir_v24i7e37600_app3.docx]

BITKZ Survey

Standard: Introduction (2 Questions)

Branch: New Branch

If

If How old are you? Not yet 18 years old Is Selected

Or How old are you? More than 24 years old Is Selected

Or How old are you? No response Thank you! Is Selected

EndSurvey:

Block: Survey (15 Questions)

Standard: Airtime Reimbursement (7 Questions)

EndSurvey:

| Page Break |  |
| --- | --- |

Start of Block: Introduction

Introductory Message
Thank you for participating in the Be in the Know Survey. The next 4 pages have 2-3 questions per page. The last page has 6 short questions. There is no right or wrong answer. Please answer as honestly as you can. Your answers will help us improve our 'Be In the Know' Application. These improvements will better serve the sexual and reproductive health needs of young people like you.
 
First we would like to know a little bit about you

Question 1 What is your age?

- Not yet 18 years old (1)
- 18 years old (2)
- 19 years old (3)
- 20 years old (4)
- 21 years old (5)
- 22 years old (6)
- 23 years old (7)
- 24 years old (8)
- More than 24 years old (9)
- No response Thank you! (10)

End of Block: Introduction

Start of Block: Survey

Question 2 What is your gender?

- Male (1)
- Female (2)
- Prefer not to answer (3)

Question 3 Which of these statements are true for you?

|  | Select Your Choice | |
| --- | --- | --- |
|  | True (1) | False (2) |
| I have heard about condoms (8) |  |  |
| If you are sexually active, using a condom correctly and consistently is the only way to prevent STDs (9) |  |  |
| Using other contraceptives with condoms is as effective as using condoms alone in preventing pregnancies (10) |  |  |
| Using two condoms is more effective than using one condom at a time (11) |  |  |
| Your healthcare provider cannot help you if your condom rips, tears, leaks or slip off (12) |  |  |
| You and your partner may need to try condoms of different types, styles, textures and sizes to find the condoms that work best for you both (13) |  |  |
| I have never had sex (14) |  |  |
| I have had sexual intercourse in the last 6 months (15) |  |  |
| The last time I had sex, I used a condom (choose False if never had sex) (16) |  |  |
| The last time I had sex, it was unplanned (choose False if never had sex) (17) |  |  |

Question 4 How strongly do you agree with the following statements:

|  | Strongly disagree (1) | Disagree (2) | Agree (3) | Strongly agree (4) |
| --- | --- | --- | --- | --- |
| I intend to have sex with my partner (28) |  |  |  |  |
| I would like to have sex to see what it is like (29) |  |  |  |  |
| I am ready to have sex if I could find a partner who would do it with me (30) |  |  |  |  |
| Most of my friends are sexually active (31) |  |  |  |  |
| Most of my friends think it is mature to practice sex at my age (32) |  |  |  |  |
| My male friends tease men who are not sexually active (33) |  |  |  |  |
| My female friends feel obliged to have sex if they accept food, drinks and entertainment (34) |  |  |  |  |
| I can decide when I can have sex (35) |  |  |  |  |
| I can decide where I can have sex (36) |  |  |  |  |
| Whether I have sex or not is entirely up to me (37) |  |  |  |  |

Question 5 People your age often feel pressure from a lot of different places and for different reasons. Thinking about yourself, in the last month how many times have you done the following because of the pressure you felt?

|  | Every time (1) | Sometimes (2) | Rarely (3) | Never (4) | I have never felt pressured to do this (5) |
| --- | --- | --- | --- | --- | --- |
| Smoked cigarettes (10) |  |  |  |  |  |
| Drunk beer or alcohol (11) |  |  |  |  |  |
| Done something sexual (12) |  |  |  |  |  |
| Did more than you had planned sexually (13) |  |  |  |  |  |
| Not used a condom (14) |  |  |  |  |  |
| Did something sexually and woke up worried about pregnancy or STIs (15) |  |  |  |  |  |

Question 6 How likely are you to do the following:

|  | Not at all Likely (1) | Somewhat Likely (2) | Very Likely (3) | Don't know (4) |
| --- | --- | --- | --- | --- |
| If you have symptoms, will you go for the STI test in the next six months? (1) |  |  |  |  |
| Have a test for HIV in the next six months? (2) |  |  |  |  |
| Use a condom the next/first time you have sex? (3) |  |  |  |  |

Question 7 Can you seek advice/ideas on sexual or reproductive health-related issues/problems from the following:

|  | I definitely can (1) | I probably can (2) | I probably cannot (3) | I definitely cannot (4) | I prefer not to answer (5) |
| --- | --- | --- | --- | --- | --- |
| Boyfriend/girlfriend (1) |  |  |  |  |  |
| Friends (2) |  |  |  |  |  |
| Someone your own age (3) |  |  |  |  |  |
| Brother/sister/cousins (4) |  |  |  |  |  |
| A parent or guardian (5) |  |  |  |  |  |
| Health care facility/worker (6) |  |  |  |  |  |
| Teacher/Professor (7) |  |  |  |  |  |
| Community leader (8) |  |  |  |  |  |
| Priest (9) |  |  |  |  |  |
| Another adult (10) |  |  |  |  |  |

Question 8 In the last month, how often have you sought advice/ideas on sexual or reproductive health related issues/problems from the following:

|  | More than once a week (1) | Once a week (2) | 1-2 times a month (3) | Never (4) | Prefer not to answer (5) |
| --- | --- | --- | --- | --- | --- |
| Facebook (1) |  |  |  |  |  |
| WhatsApp (2) |  |  |  |  |  |
| Google (3) |  |  |  |  |  |
| Instagram (4) |  |  |  |  |  |
| YouTube (5) |  |  |  |  |  |
| Be in the Know Application (6) |  |  |  |  |  |
| Friends (7) |  |  |  |  |  |
| Family (8) |  |  |  |  |  |
| Health care facility/worker (9) |  |  |  |  |  |
| Teacher/Professor (10) |  |  |  |  |  |
| Community leader (11) |  |  |  |  |  |
| Church (12) |  |  |  |  |  |
| Books/Library (13) |  |  |  |  |  |

| Page Break |  |
| --- | --- |

Question 9 How confident are you that:

|  | Not at all confident (1) | Somewhat confident (2) | Very confident (3) | Don't know (4) |
| --- | --- | --- | --- | --- |
| You can get a STI/HIV test whenever you wanted? (1) |  |  |  |  |
| You can get a condom whenever you wanted? (2) |  |  |  |  |

Question 10 The following are 14 statements about using a condom. 6 of them are correct, 8 are incorrect. Mark the 6 that are correct. You may choose not to answer this question.

- Tear along one side of the foil, being sure not to rip the condom inside (1)
- Put the condom on anytime before you ejaculate (2)
- Put the condom on when the penis is erected, before there is any contact between the penis and your partner’s body (3)
- Unroll the condom before placing on the penis (4)
- Withdraw the penis while it is still erected by holding the condom firmly in place. Remove the condom (5)
- Unroll the condom to approximately three quarters of the way down the penis (6)
- Squeeze the closed end of the condom between your forefinger and thumb and place the condom over the erected penis (7)
- Wrap the used condom back in the foil to save for the next time (8)
- Unroll the condom to the base (hair) of the penis (9)
- Apply an oil-based lubricant (i.e., oil. vaseline, lotion) (10)
- Withdraw the penis after it is no longer erected by holding the condom firmly in place. Remove the condom (11)
- Put the condom on before the penis is erected, before there is any contact between the penis and your partner’s body (12)
- Unroll the closed end of the condom keeping two inches between the end of the condom and the tip of the penis (13)
- Dispose of the used condoms (14)

Question 11 When was your most recent HIV test?

- Less than 3 months ago (1)
- 3-6 month ago (2)
- 6-12 months ago (3)
- More than 1 year ago (4)
- I have never tested for HIV (5)
- Prefer not to say (6)

Question 12 When was your most recent STI test?

- Less than 3 months ago (1)
- 3-6 month ago (2)
- 6-12 months ago (3)
- More than 1 year ago (4)
- I have never had an STI test (5)
- Prefer not to say (6)

Question 13 What is your current marital status?

- Single (1)
- Married (2)
- Divorced (3)
- Widowed/widower (4)
- Prefer not to answer (5)

Question 14 What is the last level of education you have completed?

- Less than primary school (1)
- Primary school (2)
- Secondary school (3)
- College or university (4)
- Vocational/technical school (5)

Question 15 What kind of work do you do?

- Student/trainee (1)
- Part-time job (2)
- Full-time job (3)
- Not employed (4)
- Prefer not to answer (5)

Question 16 Compared to others in your community, answer the following:

|  | How rich is your family? | | | How respected is your family? | | |
| --- | --- | --- | --- | --- | --- | --- |
|  | Very rich (1) | Average in riches (2) | Poor (3) | Very respected (1) | Respected (2) | Not respected (3) |
| Select your answer (1) |  |  |  |  |  |  |

End of Block: Survey

Start of Block: Airtime Reimbursement

Please Note
Please use the same phone number and email address for the rest of the surveys you complete as part of this research and to create your account on Be In The Know

| 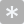 |
| --- |

Question 17 What phone number do you use for WhatsApp?

________________________________________________________________

| 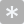 |
| --- |

Question 18 Confirm phone number do you use for WhatsApp?

________________________________________________________________

| 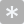 |
| --- |

Question 19 What is your Email address?

________________________________________________________________

| 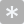 |
| --- |

Question 20 Confirm your Email address?

________________________________________________________________

Question 21 How would you like to be contacted for the next phase of the research?

- WhatsApp phone number (1)
- Email address (2)

Thank you Message
Thank you! Questions or comments?

 
Email Young Voices <youngvoicesza@gmail.com>
