# Supplementary material for: Pilot Implementation of a User-Driven, Web-Based Application Designed to Improve Sexual Health Knowledge and Communication Among Young Zambians: Mixed Methods Study
Source: J Med Internet Res. 2022 Jul 7;24(7):e37600. doi: 10.2196/37600 (PMC9305403; doi:10.2196/37600)
Supplement: Multimedia Appendix 4 [file jmir_v24i7e37600_app4.docx]

**Table 2. Population characteristics by intervention status**

| **Characteristics** | **Level** | **Comparison** | **Intervention** | **p-value** |
| --- | --- | --- | --- | --- |
|  |  | 878 | 749 |  |
|  |  | n (%) | n (%) |  |
|  |  |  |  |  |
| **Sex** |  |  |  |  |
|  | Female | 409 (46.9) | 372 (50.2) | .40 |
|  | Male | 457 (52.4) | 365 (49.3) |  |
|  | Prefer not to answer | 6 (.7) | 4 (.5) |  |
| **Age** |  |  |  |  |
|  | 18 | 44 (5.0) | 36 (4.8) | .24 |
|  | 19 | 66 (7.5) | 68 (9.1) |  |
|  | 20 | 99 (11.3) | 71 (9.5) |  |
|  | 21 | 161 (18.3) | 129 (17.2) |  |
|  | 22 | 175 (19.9) | 131 (17.5) |  |
|  | 23 | 167 (19.0) | 138 (18.4) |  |
|  | 24 | 166 (18.9) | 175 (23.4) |  |
| **Marital status** |  |  |  |  |
|  | Single | 809 (93.0) | 693 (93.3) | .97 |
|  | Married | 10 (1.1) | 9 (1.2) |  |
|  | Divorced/Widowed | 2 (.2) | 1 (.1) |  |
|  | Prefer no to answer | 49 (5.6) | 40 (5.4) |  |
| **Education level** |  |  |  |  |
|  | Less than secondary | 7 (.8) | 3 (.4) | <.01 |
|  | Secondary | 393 (45.1) | 366 (49.2) |  |
|  | Vocational/Technical | 10 (1.1) | 25 (3.4) |  |
|  | College/University | 462 (53.0) | 349 (47.0) |  |
| **Employment status** |  |  |  |  |
|  | Unemployed | 305 (35.0) | 322 (43.4) | <.01 |
|  | Student/Trainee | 424 (48.6) | 282 (38.0) |  |
|  | Part-time | 99 (11.4) | 98 (13.2) |  |
|  | Full-time | 27 (3.1) | 24 (3.2) |  |
|  | Prefer not to answer | 17 (1.9) | 16 (2.2) |  |
| **Perceived wealth** |  |  |  |  |
|  | Poor | 308 (35.4) | 159 (33.8) | .81 |
|  | Average Wealth | 558 (64.2) | 310 (65.8) |  |
|  | Very Wealthy | 3 (.3) | 2 (.4) |  |
| **Perceived respect** |  |  |  |  |
|  | Not Respected | 25 (3.0) | 12 (2.6) | .23 |
|  | Respected | 483 (58.1) | 291 (63.0) |  |
|  | Very Respected | 323 (38.9) | 159 (34.4) |  |

**Table 3. Participant sexual health history by intervention status**

| **Sexual History** | **Level** | **Comparison** | **Intervention** | **p-value** |
| --- | --- | --- | --- | --- |
|  |  | 878 | 749 |  |
|  |  | n (%) | n (%) |  |
|  |  |  |  |  |
| **Never had sex** |  |  |  |  |
|  | No | 404 (71.6) | 396 (76.0) | .10 |
|  | Yes | 160 (28.4) | 125 (24.0) |  |
| **Sex past 6 months** |  |  |  |  |
|  | No | 272 (48.1) | 216 (42.4) | .06 |
|  | Yes | 293 (51.9) | 294 (57.6) |  |
| **Condom at last sex^a^** |  |  |  |  |
|  | No | 85 (21.1) | 88 (23.9) | .37 |
|  | Yes | 317 (78.9) | 281 (76.2) |  |
| **Unplanned last sex^a^** |  |  |  |  |
|  | No | 182 (45.2) | 175 (47.4) | .65 |
|  | Yes | 221 (54.8) | 194 (52.6) |  |
| **Most recent HIV test** |  |  |  |  |
|  | Less than 3 months ago | 173 (19.9) | 168 (22.6) | .08 |
|  | 3-6 month ago | 99 (11.4) | 103 (13.9) |  |
|  | 6-12 months ago | 89 (1.2) | 50 (6.7) |  |
|  | More than 1 year ago | 307 (35.3) | 244 (32.9) |  |
|  | I have never tested for | 180 (2.7) | 160 (21.6) |  |
|  | Prefer not to say | 21 (2.4) | 17 (2.3) |  |
| **Most recent STI test** |  |  |  |  |
|  | Less than 3 months ago | 96 (11.2) | 96 (13.0) | .26 |
|  | 3-6 month ago | 55 (6.4) | 56 (7.6) |  |
|  | 6-12 months ago | 390 (45.5) | 321 (43.6) |  |
|  | More than 1 year ago | 175 (2.4) | 123 (16.7) |  |
|  | I have never had an STI | 114 (13.3) | 113 (15.4) |  |
|  | Prefer not to say | 28 (3.3) | 27 (3.7) |  |

^a^ Among those who reported ever having sex

**Table 4. Adjusted average treatment effect: “Be In The Know Zambia” intervention**

| *Variable* | *aATE* | *95% CI* | *p-value* | *Response^a^* | *Items* |
| --- | --- | --- | --- | --- | --- |
| SRH Knowledge |  |  |  |  |  |
|  | 0.12 | (-0.97, 1.2) | .83 | 2 | 6 |
| Permissiveness |  |  |  |  |  |
|  | 0.91 | (-0.3, 2.11) | .14 | 4 | 3 |
| Norms |  |  |  |  |  |
|  | -0.08 | (-2.27, 2.12) | .95 | 4 | 4 |
| Self-Efficacy |  |  |  |  |  |
|  | 0.21 | (-0.94, 1.35) | .72 | 4 | 3 |
| Resist Peer Pressure |  |  |  |  |  |
|  | 2.64 | (0.49, 4.79) | .02 | 4 | 6 |
| STI Test if symptomatic |  |  |  |  |  |
|  | 0.21 | (0.05, 0.38) | .01 | 3 | 1 |
| HIV Test in 6 months |  |  |  |  |  |
|  | 0.32 | (0.01, 0.63) | .05 | 3 | 1 |
| Use Condom next sex |  |  |  |  |  |
|  | 0.19 | (-0.6, 0.98) | .64 | 3 | 1 |
| Can Get STI Test |  |  |  |  |  |
|  | 0.18 | (-0.11, 0.46) | .22 | 3 | 1 |
| Can Get Condom |  |  |  |  |  |
|  | 0.29 | (-0.06, 0.64) | .11 | 3 | 1 |

Note: Adjusted for age, sex, marital status and employment status; UL-upper limit; LL-lower limit; STI-sexually transmitted infection; SRH-sexual reproductive health; ^a^number of responses options in scale per item; CI – confidence interval; aATE – adjusted average treatment effects.

**Table 5: Ordered regression estimates for condom-related knowledge at end line**

| *Covariate* | *Level* | *aOR* | *95% CI* | *p-value* |
| --- | --- | --- | --- | --- |
| *Intervention status* |  |  |  |  |
|  | Control | ref |  |  |
|  | Intervention | 1.35 | (1.07, 1.69) | 0.011 |
| Sex |  |  |  |  |
|  | Female | ref |  |  |
|  | Male | 0.73 | (0.59, 0.92) | 0.008 |
|  | Prefer not to answer | 0.55 | (0.05, 5.73) | 0.619 |
| Age |  |  |  |  |
|  | 18 | ref |  |  |
|  | 19 | 1.13 | (0.54, 2.37) | 0.752 |
|  | 20 | 0.91 | (0.42, 1.94) | 0.801 |
|  | 21 | 1.70 | (0.84, 3.44) | 0.141 |
|  | 22 | 1.20 | (0.6, 2.38) | 0.608 |
|  | 23 | 1.44 | (0.72, 2.9) | 0.304 |
|  | 24 | 1.38 | (0.68, 2.8) | 0.369 |
| Marital status |  |  |  |  |
|  | Single | ref |  |  |
|  | Married | 0.62 | (0.28, 1.39) | 0.246 |
|  | Divorced/Widowed | 0.61 | (0.19, 1.94) | 0.404 |
|  | Prefer not to answer | 1.06 | (0.56, 2) | 0.868 |
| Emplyment status |  |  |  |  |
|  | Unemployed | ref |  |  |
|  | Student/Trainee | 1.15 | (0.89, 1.5) | 0.281 |
|  | Part-time | 1.25 | (0.84, 1.87) | 0.268 |
|  | Full-time | 1.56 | (0.81, 3.02) | 0.185 |
|  | Prefer not to answer | 0.96 | (0.42, 2.19) | 0.923 |
| Educational attainment |  |  |  |  |
|  | Less than secondary | ref |  |  |
|  | Secondary | 1.46 | (0.4, 5.37) | 0.571 |
|  | Vocational/Technical | 0.98 | (0.24, 3.96) | 0.981 |
|  | College/University | 1.87 | (0.5, 6.98) | 0.354 |
| Preceieved wealth |  |  |  |  |
|  | Poor | ref |  |  |
|  | Average Wealth | 1.19 | (0.89, 1.58) | 0.235 |
|  | Very Wealthy | 0.75 | (0.07, 7.52) | 0.804 |
| Percieved respect |  |  |  |  |
|  | Not Respected | ref |  |  |
|  | Respected | 1.75 | (0.92, 3.36) | 0.090 |
|  | Very Respected | 1.59 | (0.82, 3.1) | 0.173 |

Note: adjusted for sex, age, marital status, employment status, educational attainment, perceived wealth, and perceived respect; aOR – adjusted odds ratio; CI - confidence interval.

**Table 6: Ordered regression estimates for condom-use knowledge at end line (N=1462)**

| *Covariate* | *Level* | *aOR* | *95% CI* | *p-value* |
| --- | --- | --- | --- | --- |
| Intervention Status |  |  |  |  |
|  | Control | ref |  |  |
|  | Intervention | 1.23 | (1.02, 1.49) | 0.03 |
| Sex |  |  |  |  |
|  | Female | ref |  |  |
|  | Male | 1.97 | (1.63, 2.39) | <.01 |
| Age |  |  |  |  |
|  | 18 | ref |  |  |
|  | 19 | 0.95 | (0.56, 1.61) | 0.84 |
|  | 20 | 0.94 | (0.56, 1.58) | 0.81 |
|  | 21 | 0.87 | (0.55, 1.37) | 0.56 |
|  | 22 | 0.97 | (0.61, 1.52) | 0.89 |
|  | 23 | 1.06 | (0.67, 1.68) | 0.79 |
|  | 24 | 1.06 | (0.67, 1.67) | 0.82 |
| Marital Status |  |  |  |  |
|  | Single | ref |  |  |
|  | Married | 1.27 | (0.62, 2.61) | 0.51 |
|  | Divorced/Widowed | 0.61 | (0.01, 31.46) | 0.80 |
|  | Prefer not to answer | 0.55 | (0.36, 0.86) | 0.01 |
| Employment Status |  |  |  |  |
|  | Unemployed | ref |  |  |
|  | Student/Trainee | 1.02 | (0.82, 1.27) | 0.83 |
|  | Part-time | 1.04 | (0.77, 1.42) | 0.80 |
|  | Full-time | 1.52 | (0.95, 2.45) | 0.08 |
|  | Prefer not to answer | 0.93 | (0.42, 2.04) | 0.85 |
| Educational attainment |  |  |  |  |
|  | Less than secondary | ref |  |  |
|  | Secondary | 1.83 | (0.47, 7.2) | 0.39 |
|  | Vocational | 1.66 | (0.37, 7.43) | 0.51 |
|  | College/University | 1.98 | (0.5, 7.82) | 0.33 |
| Perceieved wealth |  |  |  |  |
|  | Poor | ref |  |  |
|  | Average Wealth | 1.11 | (0.88, 1.39) | 0.39 |
|  | Very Wealthy | 0.56 | (0.2, 1.52) | 0.25 |
| Percieved respect |  |  |  |  |
|  | Not respected | ref |  |  |
|  | Respected | 1.12 | (0.65, 1.92) | 0.68 |
|  | Very respected | 0.90 | (0.52, 1.56) | 0.70 |

Note: adjusted for sex, age, marital status, employment status, educational attainment, perceived wealth, and perceived respect; aOR – adjusted odds ratio; CI - confidence interval.

**Table 7: Mean score for communication for sexual reproductive health advice/guidance by intervention status**

| *Person* | *Control* | *Intervention* | *p-value* |
| --- | --- | --- | --- |
|  | **Mean (SD)** | **Mean (SD)** |  |
| **Health care facility/worker** |  |  |  |
|  | 1.34 (0.64) | 1.31 (0.59) | 0.38 |
| **Boyfriend/girlfriend** |  |  |  |
|  | 1.56 (0.79) | 1.55 (0.79) | 0.97 |
| **Friends** |  |  |  |
|  | 1.60 (0.81) | 1.61 (0.8) | 0.83 |
| **Someone your own age** |  |  |  |
|  | 1.78 (0.89) | 1.74 (0.85) | 0.37 |
| **Another adult** |  |  |  |
|  | 1.89 (0.96) | 1.90 (0.94) | 0.88 |
| **Teacher/Professor** |  |  |  |
|  | 1.97 (1.05) | 1.95 (1.04) | 0.74 |
| **Brother/sister/cousins** |  |  |  |
|  | 2.12 (1.05) | 2.17 (1.07) | 0.35 |
| **A parent or guardian** |  |  |  |
|  | 2.27 (1.16) | 2.34 (1.13) | 0.21 |
| **Community leader** |  |  |  |
|  | 2.33 (1.12) | 2.3 (1.13) | 0.68 |
| **Priest** |  |  |  |
|  | 2.40 (1.16) | 2.52 (1.15) | 0.04 |

Note: Mean according to 1-4 scale where 1-definitely can communicate, 2-probably can communicate, 3-probably cannot communicate, 4-definitely cannot communicate, i.e., higher mean values correspond to decreased comfort communicating sexual and reproductive health issues/problems; p-value derived from t-test; SD – standard deviation.

**Table 8: User rating of the BITKZ app**

| *Factor* | *Level* | *Responses*  *N (%)* |
| --- | --- | --- |
| N |  | 637 |
| **Overall Rating** |  |  |
|  | Excellent | 359 (56.4) |
|  | Good | 237 (37.2) |
|  | Satisfactory | 28 (4.4) |
|  | Poor | 6 (0.9) |
|  | Fail | 2 (0.3) |
|  | No response | 5 (0.8) |
| **Aesthetics** |  |  |
| Looks good |  |  |
|  | Not at all | 8 (1.3) |
|  | Quite | 184 (28.9) |
|  | Very | 445 (69.9) |
|  | No response | 0 (0.0) |
| Like graphics |  |  |
|  | Not at all | 7 (1.1) |
|  | Quite | 197 (30.9) |
|  | Very | 425 (66.7) |
|  | No response | 8 (1.3) |
| Like arrangement |  |  |
|  | Not at all | 8 (1.3) |
|  | Quite | 127 (19.9) |
|  | Very | 485 (76.1) |
|  | No response | 17 (2.7) |
| **Engagement** |  |  |
| Age appropriate |  |  |
|  | Not at all | 2 (0.3) |
|  | Quite | 88 (13.8) |
|  | Very | 540 (84.8) |
|  | No response | 7 (1.1) |
| Interesting |  |  |
|  | Not at all | 4 (0.6) |
|  | Quite | 108 (17.0) |
|  | Very | 511 (80.2) |
|  | No response | 14 (2.2) |
| Entertaining |  |  |
|  | Not at all | 7 (1.1) |
|  | Quite | 189 (29.7) |
|  | Very | 426 (66.9) |
|  | No response | 15 (2.4) |
| Personalizable |  |  |
|  | Not at all | 37 (5.8) |
|  | Quite | 286 (44.9) |
|  | Very | 302 (47.4) |
|  | No response | 12 (1.9) |
| Interactiveness |  |  |
|  | Not at all | 14 (2.2) |
|  | Quite | 180 (28.3) |
|  | Very | 432 (67.8) |
|  | No response | 11 (1.7) |
| More likely to use if in own language |  |  |
|  | Not at all | 69 (10.8) |
|  | Quite | 172 (27.0) |
|  | Very | 386 (60.6) |
|  | No response | 10 (1.6) |
| More likely to share if in own language |  |  |
|  | Not at all | 58 (9.1) |
|  | Quite | 208 (32.7) |
|  | Very | 360 (56.5) |
|  | No response | 11 (1.7) |
| **Functionality** |  |  |
| Worked well |  |  |
|  | Not at all | 13 (2.0) |
|  | Quite | 154 (24.2) |
|  | Very | 468 (73.5) |
|  | No response | 2 (0.3) |
| Easy to use |  |  |
|  | Not at all | 12 (1.9) |
|  | Quite | 116 (18.2) |
|  | Very | 487 (76.5) |
|  | No response | 22 (3.5) |
| Easy to move between content |  |  |
|  | Not at all | 18 (2.8) |
|  | Quite | 159 (25.0) |
|  | Very | 439 (68.9) |
|  | No response | 21 (3.3) |
| **Information** |  |  |
| Useful |  |  |
|  | Not at all | 10 (1.6) |
|  | Quite | 60 (9.4) |
|  | Very | 564 (88.5) |
|  | No response | 3 (0.5) |
| Easy to understand |  |  |
|  | Not at all | 8 (1.3) |
|  | Quite | 76 (11.9) |
|  | Very | 545 (85.6) |
|  | No response | 8 (1.3) |
| High quality of content |  |  |
|  | Not at all | 6 (0.9) |
|  | Quite | 164 (25.7) |
|  | Very | 460 (72.2) |
|  | No response | 7 (1.1) |
| Vastness of quantity of content |  |  |
|  | Not at all | 16 (2.5) |
|  | Quite | 282 (44.3) |
|  | Very | 328 (51.5) |
|  | No response | 11 (1.7) |
| Believable content |  |  |
|  | Not at all | 5 (0.8) |
|  | Quite | 135 (21.2) |
|  | Very | 479 (75.2) |
|  | No response | 18 (2.8) |
| **Sharability** |  |  |
| Likely will share with friends in future |  |  |
|  | Not at all | 4 (0.6) |
|  | Quite | 84 (13.2) |
|  | Very | 542 (85.1) |
|  | No response | 7 (1.1) |
| Likely will use app again |  |  |
|  | Not at all | 3 (0.5) |
|  | Quite | 81 (12.7) |
|  | Very | 538 (84.5) |
|  | No response | 15 (2.4) |
| Likely will recommend app |  |  |
|  | Not at all | 3 (0.5) |
|  | Quite | 80 (12.6) |
|  | Very | 543 (85.2) |
|  | No response | 11 (1.7) |
| Likely will pay for app |  |  |
|  | Not at all | 70 (11.0) |
|  | Quite | 349 (54.8) |
|  | Very | 205 (32.2) |
|  | No response | 13 (2.0) |
