# Supplementary material for: Pilot Implementation of a User-Driven, Web-Based Application Designed to Improve Sexual Health Knowledge and Communication Among Young Zambians: Mixed Methods Study
Source: J Med Internet Res. 2022 Jul 7;24(7):e37600. doi: 10.2196/37600 (PMC9305403; doi:10.2196/37600)
Supplement: Multimedia Appendix 5 [file jmir_v24i7e37600_app5.docx]

**Textbox 1. User’s statements on effect of BITKZ on peer pressure**

| ‘So the app just made it easy for me to actually put things in line. To say, ‘Okay, these are the things that my friends expect me to do, but I won't be able to do it because it doesn’t sit right with me.’ So, that's handling all of these pressures that come from outside. I feel like that also helps.’ - Female, 23 years old  ‘Where there is that peer pressure, you try to debate with your mind on what to do or what not to do, will it hurt you ...’ - Female, 23 years old  ‘Most memorable? Aah it’s about some situations where the peer to peer situation? Yeah. Because there was a passage I read about, also about drugs, drug abuse something like that, and yeah, so I was excited about this, because it was telling me on how I can prevent peer pressure.’ – Male, 19 years old  ‘There are some things I've done. So what I thought was I should have come across this application earlier; I wouldn't have done things that I did in my past. It has also helped me to stay away from a lot of things.  [I: Okay, things like what?]  ‘Things like the sexual things, for yes, I almost started taking alcohol because of peer pressure. And all the sexual health, the time I came across this application, it mentioned a lot of things about that. It has helped me to keep myself away from such things.'  – Male, 22 years old  ‘Mmmm this information can be useful to me because um, being young there is a lot of peer pressure … Sometimes there are complicated situations because we are young and we don’t know how to handle the situations … We even have pressure, where they what, what can I say? Where the solution is just simple, you are having pressure because you don’t have a lot of information about sex or relationships … Because us when we are in relationships …We seem to think like okay, okay like, we think we should help others, like, we should impress our girlfriends.  - Male, 24 years old |
| --- |

**Textbox 2. User’s statements on effect of BITKZ on condom/contraceptive-related knowledge**

| ‘It (App) was sharing stuff about how to use condoms, the right use of how to operate it, what to do if one had sex without a condom, I think it was very educative on the condoms, STI aspect.’ - Female, 21 years old  ‘What I was thinking of is how I had been putting on the condom and how it has to be put on, they are very different.’ - Male, 20 years old  I never knew doubling up a condom makes everything unprotected, coz for me, I thought, “When you double up, double shield!” So it’s funny that I didn’t … actually I never thought until I read about it, and I did some background search and found out it was true. - Male, 21years old  I thought like the condoms are only made for like for old people or those people that are in marriages, but then from there, I was able to know that everyone, anyone can use a condom. You can be you, can be young or old, anyone can use a condom.   - Male, 22 years old   ‘I had the fear of using it [female condom] before the app. But now that they have illustrated how to use it, I am comfortable thinking about it.’   - Female, 20 years old participant   There are different contraceptive methods you can use if you want to prevent pregnancy ... There is an injection. There's also … I don’t know what it's called but they put something - it’s an implant. Yeah, and there are also in case … you didn’t use protection ... there are emergency pills like morning after.  - Female, 22 years old |
| --- |

**Textbox 3. Women’s statements on effect of BITKZ on partner communication**

| Most of us girls are not free in relationships like to say what we want. We are not free to make choices (…) It is kind of like the man has to make the choices for us. - Female, 24 years old  So the information I found useful was that you need to get free with your partner. If you don't feel like what the other person is saying, you should have a free mind to say no; or yes if you agree. Then your partner will have to respect the decision that you made. - Female, 19 years old  For me I shared it with my boyfriend … I told him that there is this App to go through, maybe because there are certain things that I am not comfortable telling him. So, after reading on his own definitely he had the idea, was also learning here and there, and we talk about protection now. - Female, 24 years old  It was amazing, like the answer that I got from the app is the answer that he [boyfriend] gave me, like actually it’s supposed to come from both of us, we have to plan for this together so that we can also be free. If we are having sex, we know that we are having a protected one. ’ - Female, 22 years old, virgin |
| --- |
